# Assessing ChatGPT 4.0’s test performance and clinical diagnostic accuracy on USMLE STEP 2 CK and clinical case reports

**DOI:** 10.1038/s41598-024-58760-x

**Published:** 2024-04-23

**Authors:** Allen Shieh, Brandon Tran, Gene He, Mudit Kumar, Jason A. Freed, Priyanka Majety

**Affiliations:** 1 Virginia Commonwealth University School of Medicine, Richmond, VA USA; 2 Division of Child and Adolescent Psychiatry, Department of Psychiatry, Virginia Commonwealth University, Richmond, VA USA; 3 Division of Hematology and Hematologic Malignancies, Department of Internal Medicine, Beth Israel Deaconess Medical Center, Boston, MA USA; 4 Division of Endocrinology, Diabetes and Metabolism, Department of Internal Medicine, Virginia Commonwealth University, Richmond, VA USA

**Keywords:** ChatGPT 4, USMLE, Case reports, Diagnostic accuracy, Diagnosis, Health occupations, Medical research

## Abstract

While there is data assessing the test performance of artificial intelligence (AI) chatbots, including the Generative Pre-trained Transformer 4.0 (GPT 4) chatbot (ChatGPT 4.0), there is scarce data on its diagnostic accuracy of clinical cases. We assessed the large language model (LLM), ChatGPT 4.0, on its ability to answer questions from the United States Medical Licensing Exam (USMLE) Step 2, as well as its ability to generate a differential diagnosis based on corresponding clinical vignettes from published case reports. A total of 109 Step 2 Clinical Knowledge (CK) practice questions were inputted into both ChatGPT 3.5 and ChatGPT 4.0, asking ChatGPT to pick the correct answer. Compared to its previous version, ChatGPT 3.5, we found improved accuracy of ChatGPT 4.0 when answering these questions, from 47.7 to 87.2% (*p* = 0.035) respectively. Utilizing the topics tested on Step 2 CK questions, we additionally found 63 corresponding published case report vignettes and asked ChatGPT 4.0 to come up with its top three differential diagnosis. ChatGPT 4.0 accurately created a shortlist of differential diagnoses in 74.6% of the 63 case reports (74.6%). We analyzed ChatGPT 4.0’s confidence in its diagnosis by asking it to rank its top three differentials from most to least likely. Out of the 47 correct diagnoses, 33 were the first (70.2%) on the differential diagnosis list, 11 were second (23.4%), and three were third (6.4%). Our study shows the continued iterative improvement in ChatGPT’s ability to answer standardized USMLE questions accurately and provides insights into ChatGPT’s clinical diagnostic accuracy.

## Introduction

Artificial Intelligence (AI) has grown to influence multiple professional sectors. The applications of AI are broad and can improve the efficiency of complex tasks. Through machine learning, AI-based programs develop working code, create unique music, and even diagnose complex diseases based on anamnesis, lab results, radiological images, or pathologic results^1–4^. However, significant work needs to be done to fulfill the promises its application has in the field of medicine.

Based on a Large Language Model (LLMs) and trained on copious data to reconstruct original outputs, AI programs such as ChatGPT are in their infancy with little research existing about its functions and applications in healthcare settings. Little is also known about ChatGPT’s evolution in performance across multiple iterations. ChatGPT 4.0, the most current iteration of AI LLMs, boasts numerous features such as speedy response times, visual media creation via its DALLE counterpart, and soon-to-be exalted image recognition.

One area of research for ChatGPT in healthcare has been primarily on its ability to answer questions from various standardized medical examinations. Gilson et al.^5^ found an accuracy of 60% across both the United States Medical Licensing Exam (USMLE) STEP 1 and STEP 2. Kung et al.^6^ found similar results and additionally tested ChatGPT’s capabilities further by analyzing its logic through a 2–3 physician grading system. This grading system assessed its responses in terms of logic, validity, and non-obvious insights in order to understand how ChatGPT could be used and understood as a tool for medical students.

In the realm of medical sciences, ChatGPT’s ability to “understand and reason” has been a point of controversy. Although there are claims that ChatGPT is able to deductively reason and have clear trains of thought, others have found the chatbot is at risk of artificial hallucinations, which are factual errors that are derived from unknown or fake sources. When ChatGPT is asked to cite its sources for its claims, the sources appear to be real, but when searched up, do not exist^5,7^.

The integration of AI in healthcare, while having the potential to help clinicians, also brings forth several ethical concerns, including the protection of patient privacy and data security, addressing inherent biases in AI algorithms, ensuring transparency, maintaining patient autonomy and informed consent, preventing misinformation, and preserving the quality of the patient-provider relationship^8^. Májovský et al.^9^ reported that users can easily misuse ChatGPT to fabricate seemingly authentic scientific manuscripts that appear properly formatted, compromising the integrity of academic medicine.

There are limited studies on its performance in real-world clinical scenarios. A recent study by Kanjee et al.^10^ showed that ChatGPT 4.0 provided the correct diagnosis in its differential in 64% of challenging cases, using the New England Journal of Medicine (NEJM) clinicopathologic conferences. Other studies compared ChatGPT to physicians on handling realistic clinical settings, such as that of Hirosawa et al.^11^ who examined how well ChatGPT could generate differential diagnoses for common chief complaints. They found that ChatGPT yielded a correct diagnosis over 90% of the time, creating a shortlist of top 10 diagnoses. However, when compared with physicians on a list of three or five differentials, it performed significantly worse, with the most common error being the incorrect order of priority in differentials. Overall, its logic and by extension, clinical reasoning was considered reasonably sound in greater than 90% of responses^11^.

There is a deficiency of studies comparing the performance of ChatGPT 3.5 vs 4^12^, especially in healthcare. Massey et al. compared the performance of ChatGPT 3.5, ChatGPT 4.0 and orthopedic residents on orthopedic assessment examinations.

These mixed experiences with the use of ChatGPT in difficult applications, such as medicine, warrants more research to characterize its ability to logically and ethically reason through complex medical problems. We aimed to compare ChatGPT 4.0’s performance in accurately answering board-style questions with ChatGPT 3.5 and further evaluate its potential value as a tool for diagnosis, workup, management, and follow up based on published clinical case reports.

## Methods

The USMLE provides 120 free Step 2 CK practice questions on the official USMLE website. Questions stemmed from a June 2022 sample exam release date, which was outside of the training samples for ChatGPT. The 120 questions were tabulated into a spreadsheet, and filtered for any image-based questions, which are incompatible with ChatGPT 3.5 and 4.0. These sample questions were compiled and further encoded.

**Figure 1:**
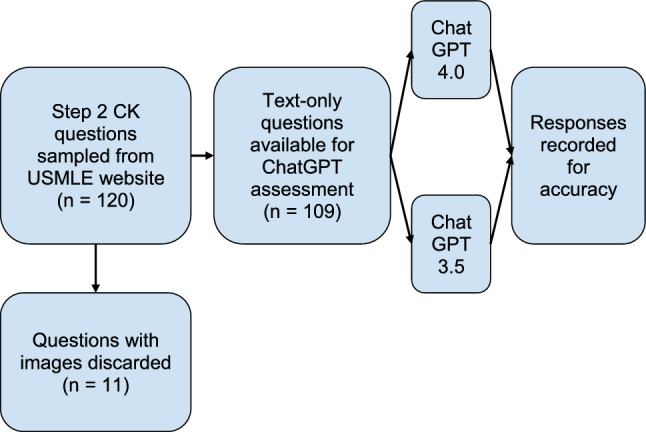
Comparing ChatGPT 4.0’s performance on the USMLE Step 2 practice exam to its previous version, ChatGPT 3.5.

Questions were categorized based on the type of question asked. Categories included diagnosis, next step in management, non-medical, best treatment, and prevention. These categories were determined based on the final question stem in the vignette. Examples included “What is the diagnosis?”, “What is the next best step in management?”, “Which of the following pharmacotherapies is appropriate?” or “Which of the following would have prevented…?”. For non-medical questions, any questions covering topics such as statistics were considered non-medical. After compilation and encoding, we had 109 questions suitable for input (Fig. 1). These questions were input verbatim into ChatGPT 3.5 and ChatGPT 4.0, with the final question asking it to choose the best answer from the multiple-choice section. A new chat session was created with each new question in order to reduce memory retention bias. Outputs were marked as either correct or incorrect based on the answer key provided by USMLE.

**Figure 2:**
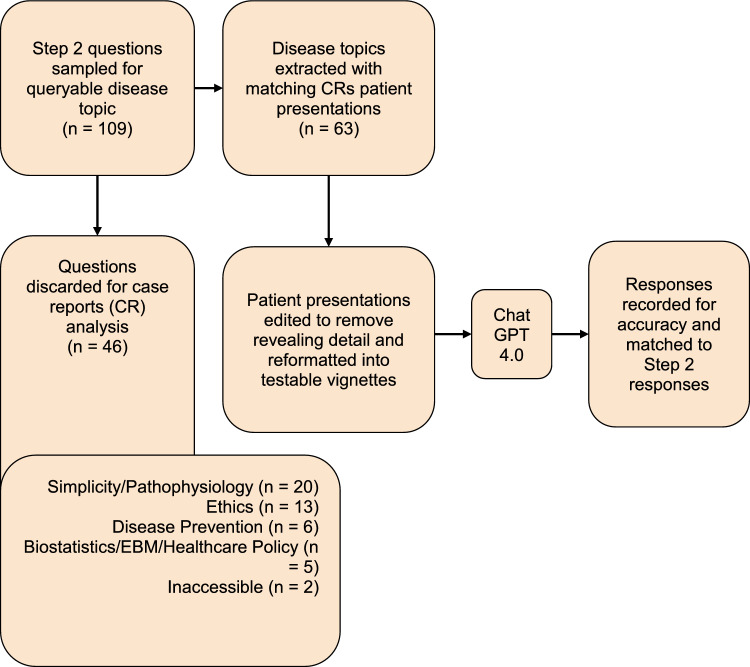
Assessing ChatGPT 4.0’s ability to generate accurate differential diagnoses when fed published case reports (CR).

For every one of the 109 questions that were initially input into ChatGPT, we queried case reports about the tested disease processes from Pubmed/MEDLINE (Fig. 2). Cases were categorized as pre- or post-2021 to parse out which case reports could have been included in the ChatGPT training set. Our primary question for these case reports was centered on generating a differential diagnosis. Consequently, any topic or question where we could not apply this strategy was excluded from the analysis due to a lack of available case reports. These scenarios included portrayal of high prevalence pathophysiology such as bee stings that are typically not case reportable, ethical decision making, questions based on USPSTF screening recommendations or similar guidelines, interpretation of medical literature, biostatistical calculations, or inaccessibility.

**Figure 3:**
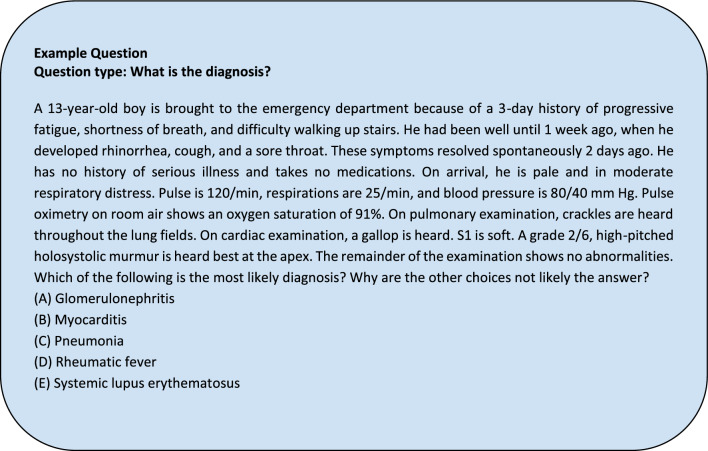

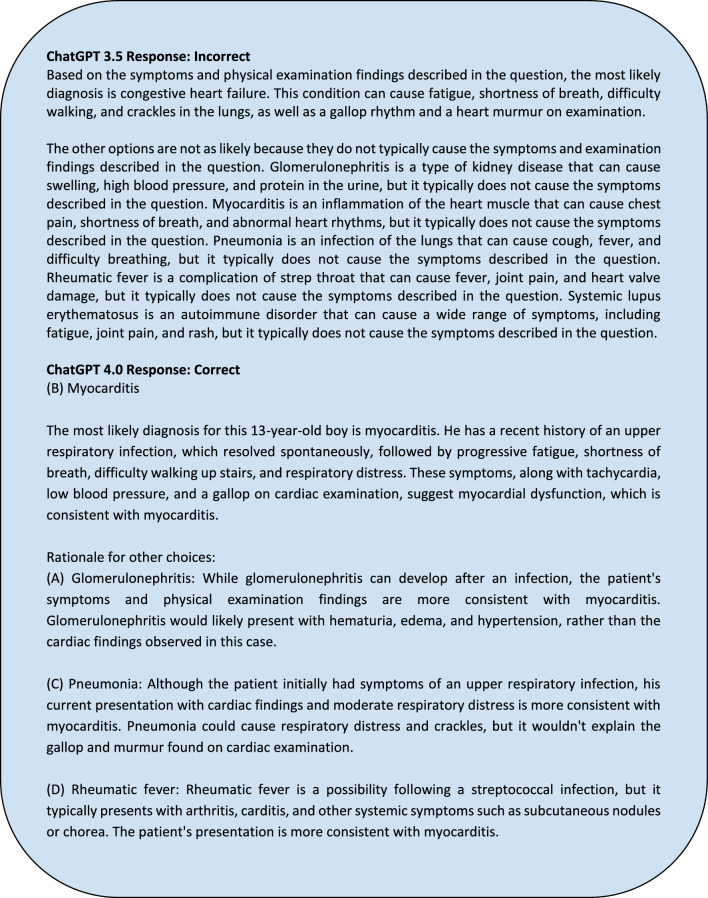
(**a**) Example USMLE-style question prompted to ChatGPT. (**b**): Example ChatGPT response to the USMLE-style question.

**Figure 4:**
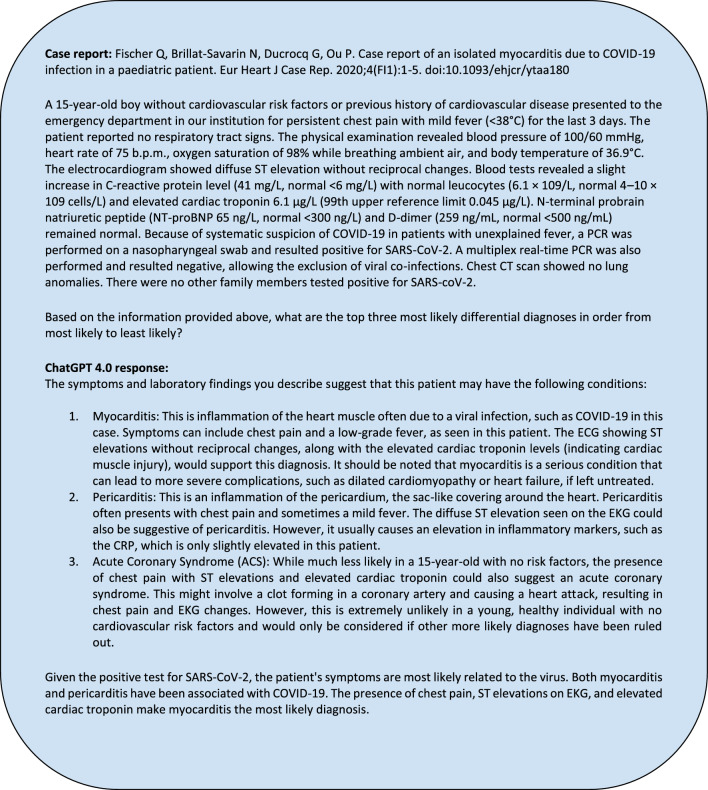
Example of ChatGPT 4.0’s responses when assessing its ability to generate differential diagnoses when a corresponding case report details were inputted.

Of the 109 questions we fed into ChatGPT, 63 case report (CR) vignettes were paired with 63 standardized sample question vignettes based on matching disease topics. From these case reports, the history section of the case report was parsed by the authors. For instance, in a case report on septic arthritis, any synovial fluid analysis or imaging of the infected joint was excluded from the final input into ChatGPT 4.0. Examples of prompts inputted are in Appendix A. After parsing through the case report to ensure only pertinent, non-diagnostic information would be inputted, we entered it into ChatGPT with the additional prompt “Based on the provided information above, what are the top three most likely differential diagnoses in order from most to least likely?” appended. New chat sessions were created with each case report to reduce memory retention bias. An example of an input and output for a question is shown in Figs. 3a,b, and 4.

We also analyzed ChatGPT 4.0’s confidence in its diagnosis by asking it to rank its top three differentials from most to least likely. Responses were recorded based on their correctness, as well as whether or not ChatGPT had the correct diagnosis on the 1st, 2nd, or 3rd differential. A response was considered “correct” if either one of the differentials included the presumed diagnosis from the case report.

## Statistical analysis

The analysis was performed using IBM SPSS Statistics version 29.0.0.0. The chi-squared test was used as a non-parametric statistical method to determine associations between categorical variables. These variables were the accuracies between ChatGPT 3.5 and 4.0 responses to STEP2 CK. We additionally examined the association between ChatGPT 4.0’s performance on case reports matched to the corresponding STEP 2 CK question. A p-value of < 0.05 was used as a determination of statistical significance.

## Results

### ChatGPT 3.5 vs. ChatGPT 4.0

Out of the 109 questions inputted into ChatGPT, 52 (47.7%) and 95 (87.2%) of the questions were answered correctly with ChatGPT 3.5 and ChatGPT 4.0, respectively (p 0.35). We then selected ChatGPT 4.0 to advance to the next part of data collection and analysis (i.e., case report testing) because of its 40% lead in test-taking accuracy across all categories (Table 1).

**Table 1:** Comparison of accuracy of ChatGPT4.0 based on multiple choice question categories.

Question categories	# of Questions	ChatGPT 3.5		ChatGPT 4.0		*P*-value
		# of Incorrect answers	Accuracy in %	# of Incorrect answers	Accuracy in %	
Diagnosis	33	15	54.54	5	85.85	
Next step in management	29	18	37.93	3	89.66	
Non-medical	19	6	68.42	1	94.74	
Best treatment	15	11	26.67	3	80.00	
Best prevention	10	6	40.00	2	80.00	
Total	109	57	47.7	14	87.2	0.035

### Clinical accuracy—case reports

Of the 109 questions, 63 had disease topics with pertinent case reports. ChatGPT 4.0 correctly identified the diagnosis in 47 out of 63 matched case report vignettes (74.6% accuracy) compared to 54 out of 63 in the corresponding standardized sample question vignettes on the same diseases (85.7% accuracy) (Table 2). There was also a statistically significant association in diagnostic accuracy between ChatGPT 4.0’s assessment of standardized sample question vignettes and case report vignettes (*p* < 0.002). Of the 63 case reports, 54 were published pre-2021, and 9 were published post-2021. There was no statistical difference in the accuracy between these 2 groups (Supplementary table S1).

**Table 2:** Comparison of diagnostic accuracy between standardized sample question vignettes and Case Report (CR) vignettes matched by shared disease topic.

	Standardized vignettes	Case report vignettes	*p-value*
Total questions	63	63	–
Total Correct	54 (85.7%)	47 (74.6%)	0.002
# Correct on 1st differential	–	33 (70.2%)	
# Correct on 2nd Differential	–	11 (23.4%)	
# Correct on 3rd Differential	–	3 (6.4%)	

Percent accuracies are denoted in parentheses.

### Confidence

We also analyzed ChatGPT 4.0’s confidence in its diagnosis by asking it to rank its top three differentials from most to least likely. Out of the 47 correct diagnoses, 33 were the first (70.2%) on the differential diagnosis list, 11 were second (23.4%), and three were third (6.4%). Sixteen case reports did not return any correct diagnoses in the top three differentials (Table 2).

## Discussion

The generative AI model, ChatGPT 4.0 continues to significantly improve its performance on the standardized sample questions compared to its previous versions. It provided the correct diagnosis in its differential in 74.6% of the corresponding clinical case reports and as its top diagnosis in 70.2%. Our findings compare favorably with existing studies and may suggest improved confidence in clinical diagnosis^10^.

This leap in reasoning and understanding in medicine extends beyond other fields and exams. OpenAI claims ChatGPT 4.0 is capable of passing the bar exam, LSAT, and GRE among other standardized exams^13^. Its ability to answer academic questions in multiple-choice format is consistently at or above passing scores and continues to trend upwards.

After a limited qualitative analysis of ChatGPT 4.0’s response justifications, we found that case reports featuring incredibly rare diseases or diseases masquerading as another generated most of the incorrect diagnoses. Rare diseases tended to have subtle initial presentations, with patients presenting with sequelae of the primary defect. ChatGPT 4.0 would diagnose the presentation but would fail to suggest an underlying cause. One example of this mistake involves a case report on the VACTERL association, which is an acronym for the rare co-occurrence of congenital abnormalities including vertebral defects (V), anorectal malformations (A), cardiac defects (C), tracheoesophageal fistula with or without esophageal atresia (TE), renal malformations (R), and limb defects (L). The infant’s presentation was fairly non-specific, consisting primarily of respiratory issues, and as a result, ChatGPT’s differential included pneumonia, bronchiectasis, and tuberculosis, all of which failed to understand the root cause of the infant's symptoms. While ChatGPT 4.0 is able to accurately follow along relatively simple and straightforward cases, we think it fails to reliably understand nuanced cases with underlying issues masked by initial presentations.

Our study has several limitations. We utilized published case reports to assess ChatGPT’s diagnostic accuracy. Though case reports are important contributors to medical knowledge and help remind practitioners about clinical conundrums or rare presentations of diseases, they are also uncommon and do not represent the vast majority of patient presentations. This limits the generalizability of our study. Another shortcoming of our study is the lack of a human comparator arm. Having physician comparators would help us better understand the practical nature of AI Chatbots in medical workflow. It would also help assess if the decision-making between a physician and ChatGPT would differ on a certain patient, and why. Our study has a small sample size of 63 case reports and we acknowledge that this could have potentially affected the strength of our results. Our study design limits the applicability of its medical reasoning to real-life medical conundrums and is lacking in exploring decisions behind management, work-up, discharge planning, and follow-up. It only examined ChatGPT’s ability as a diagnostic tool. Though this may help drive certain clinical decisions surrounding what diagnostic labs or imaging to order, our study does not explore how ChatGPT can guide symptomatic, definitive, or maintenance treatment of patients. Its ability to augment real-life decision-making remains addressed. Additionally, most of our cases predated 2021, meaning it may have been included in the initial training set for ChatGPT. This brings up the question as to whether or not ChatGPT is regurgitating information it has seen before or truly generating a unique response to our prompts.

Regardless of these limitations, we believe these results add to the existing literature in understanding its role as a tool in clinical diagnosis. We envision that it could be used as an adjunctive tool for medical trainees and healthcare providers. ChatGPT’s high but sub-optimal accuracy limits its clinical applicability but shows promise in academia. Possible situations of its applicability include personalized, conversational explanations when learning why certain answer choices are correct and others are incorrect in standardized examinations, outlining summaries of published literature with diagnostic rationale, and work up^2,14^.

The integration of AI into medicine, especially in clinical settings, brings both transformative potential and ethical challenges. While AI has the potential to enhance efficiency as machine learning algorithms can analyze vast datasets, such as medical imaging or genetic information, more rapidly, the deployment of AI in medicine raises ethical concerns that must be meticulously addressed. Privacy and data security are paramount, as AI systems require access to sensitive patient information. There's also the risk of algorithmic bias, where AI models might perpetuate or even exacerbate existing disparities in healthcare due to biased training data or algorithms. Ensuring transparency and explainability in AI-driven decisions is critical to maintaining trust and accountability in patient care. Furthermore, there's a need to consider the impact on the physician–patient relationship, as the introduction of AI could depersonalize care or shift the dynamics of clinical decision-making. Balancing the immense benefits of AI in medicine with these ethical considerations is crucial for its responsible and effective integration into healthcare.

Future larger-scale studies should investigate its ability to suggest up-to-date guideline-directed management strategies in clinical situations, which will further evaluate its utility as a clinical management tool. It would be beneficial evaluate ChatGPT’s performance in real-life clinical scenarios faced by clinicians daily with physician comparators. This will help understand the limitations of LLM in medicine and better define its role in practical clinical medicine. With the results of this and future studies ChatGPT could become a helpful adjunctive tool for students to learn evidence-based medicine via a patient-based approach as well as become an adjunctive clinical decision-making tool.

## Conclusion

We showed ChatGPT’s improvement in test-taking accuracy between versions 3.5 and 4.0, as well as demonstrated ChatGPT’s diagnostic accuracy on patient presentations documented in case reports. These results show the gradual and continual improvement in AI technology in being implemented into the workflow of medicine. Although there are several examples of the AI technology being implemented to conduct various medical tasks^15,16^, further research assessing AI’s performance with data from real-world patient encounters is needed to better characterize its role as a reliable tool for adjunct clinical diagnosis.

### Supplementary Information

Supplementary Information 1.

Supplementary Table S1.

## Data Availability

Data is provided within the manuscript or supplementary information files.
