# Supplementary material for: Assessing ChatGPT 4.0’s test performance and clinical diagnostic accuracy on USMLE STEP 2 CK and clinical case reports
Source: Sci Rep. 2024 Apr 23;14:9330. doi: 10.1038/s41598-024-58760-x (PMC11039662; doi:10.1038/s41598-024-58760-x)
Supplement: Supplementary file 1 — Supplementary Information 1. [file 41598_2024_58760_MOESM1_ESM.docx]

1.2 - Thobe N, Pilger P, Jones MP. Primary hypothyroidism masquerading as hepatic encephalopathy: case report and review of the literature. Postgrad Med J. 2000;76(897):424-426. doi:10.1136/pmj.76.897.424

A previously healthy, functional 74 year old woman was admitted in coma after being found unresponsive in her flat. Several days before she had fallen and struck her head without loss of consciousness. Since then, she had exhibited slurred speech and did not seem herself. Her past medical history as reported at the time of admission was remarkable for well compensated hepatitis C of approximately 20 years' duration. A search of the patient's flat found no prescription medication. She neither smoked nor drank.

Examination revealed a thin female in coma with shallow, irregular respirations. Her pulse was 95 beats/min, blood pressure was 120/50 mm Hg, and respirations were 6–8/min. A healing contusion was noted on her forehead. Examination of the abdomen showed the liver to be of normal span with a firm edge. There was no splenomegaly or ascites noted. There was no oedema or spider angiomas, but palmar erythema was noted. Neurologically, she was unresponsive. Her Glasgow coma score was 3.

A complete blood count, electrolytes, urinalysis, and prothrombin time were within normal limits. Results of liver chemistry were (normal limits): aspartate aminotransferase (AST) 156 U/L (11–35), alanine aminotransferase (ALT) 53 U/l (7–46), alkaline phosphatase 113 U/l (46–139), total bilirubin 37.6 μmol/l (3.4–20.5), direct bilirubin 10.3 μmol/l (0–5.1), and albumin 34 g/l (34–48). The serum ammonia concentration was 124 μmol/l (10–47). Computed tomography of the head revealed no evidence of intracranial hemorrhage or cerebrovascular accident. Lumbar puncture revealed a normal opening pressure, no gross or microscopic blood, and normal concentrations of glucose and protein. An echocardiogram showed no valvular abnormalities, pericardial effusion, thrombus, or vegetations. Ultrasound examination of the abdomen disclosed a small, echogenic liver and splenomegaly. An electroencephalogram revealed severe generalized slowing with triphasic waves.

**What is the differential diagnosis? What are the best three next steps in management?**

*A diagnosis of hepatic coma was made and the patient started on lactulose. Within 24 hours, the ammonia level decreased to 52 μmol/l, but her mental status was unchanged. Because of the poor clinical response, other aetiologies for metabolic encephalopathy were sought. A morning cortisol was normal and a serum toxin screen was negative. Her serum thyroid stimulating hormone was 104 mU/l (0.47–6.90).*

**Given this additional information, what is the differential diagnosis? What are the best three next steps in management?**

2.2 - Alam V, Nanzer AM. Eosinophilic granulomatosis with polyangiitis: case report and literature review. Breathe (Sheff). 2022;18(4):220170. doi:10.1183/20734735.0170-2022

A 41-year-old female patient was referred to the severe asthma clinic with a diagnosis of Samter's triad (asthma, aspirin-exacerbated respiratory disease and rhinosinusitis) made 18 years ago. Despite treatment with and optimal adherence to high dose inhaled corticosteroids/long-acting β-agonist and long-acting muscarinic antagonist inhalers and montelukast, she had suffered from recurrent exacerbations needing three courses of oral corticosteroid therapy in the preceding 12 months.

At her first clinic review she had a peripheral blood eosinophil count of 0.9×109 cells per L, total IgE 55 kU·L−1 with specific IgE positive to house dust mite 22.5 kUA·L−1. She had a forced expiratory volume in 1 s (FEV1) of 2.56 L (84%) and evidence of airway inflammation based on the fraction of exhaled nitric oxide (FENO) of 122 ppb. On computer tomography (CT) images of the chest there was mild bronchial wall thickening, a CT of the sinuses showed mild to moderate mucosal thickening.

3.2 - Hu TC, Ker CR, Long CY. Urine retention caused by urethral prolapse mimicking uterine prolapse. Taiwan J Obstet Gynecol. 2020;59(1):165-166. doi:10.1016/j.tjog.2019.11.029

A 64-year-old woman presented with urinary retention, painful vaginal bleeding and a protruding mass for a few hours. According to patient herself, she had found mild tissue protruding from private parts one year ago. Obstetric history included two vaginal deliveries and one ectopic pregnancy. She had reached menopause at the age of 53 years. She denied other systemic disease. Abdominal ultrasound showed no significant findings. Pelvic examination revealed a reddish round doughnut-shaped mass (3 × 2 cm) located at introitus, resembling uterine cervix. However, the protruding mass could not be repositioned and the patient felt uncomfortable with attempts to reduce the presumed prolapse. A Foley catheter was inserted to confirm circumferential eversion of distal urethra.

4.2 - Soto SM, Chen J, Izakovich T, I Genin D. METHIMAZOLE-INDUCED AGRANULOCYTOSIS: A CASE REPORT AND LITERATURE REVIEW. Chest. 2022;162(4):A905. doi:10.1016/j.chest.2022.08.714

43 yo woman with a history of recently diagnosed Graves disease who presented to the emergency room with fever, generalized weakness, and decreased oral intake 42 days after starting Methimazole. Upon admission, CBC revealed leukocytes of 1.26 k/uL, with an absolute neutrophil count of 0. CTAP with mesenteric and para- aortic adenopathy.

5.2 - Taylor HL, Wellman LL, Conran RM. Educational Case: Neisseria Meningitis. Acad Pathol. 2021;8:23742895211043244. doi:10.1177/23742895211043244

An 18-year-old college student who lives in the campus dormitory presents to the emergency department (ED) with fever and a stiff neck that began a few hours earlier. He also describes a headache and nausea that started at the same time. Past medical history is noncontributory. He has not seen a physician since the age of 13. His social history reveals recreational marijuana use and periodic alcohol ingestion.

Physical examination reveals a well-nourished individual who is confused and in moderate distress. Vital signs are temperature 103 °F, heart rate 120 beats per minute, blood pressure 95/60 mm Hg, and respiratory rate of 20 breaths per minute. Cardiac examination reveals a normal S1 and S2 with regular rhythm without murmurs, heaves, or gallops. Lungs are clear to auscultation. The abdomen is soft, nontender with positive bowel sounds and no masses on palpation. A petechial rash is noted on his trunk. His skin is cold and clammy. Kernig and Brudzinski signs are positive. He is oriented to person and place but not time and has no focal neurological deficits. The remaining examination is within normal limits.

7.2 - Rincón Franco S, Uriel M, Rodríguez LM, Romero Infante XC. Preventive measures to avoid vertical transmission in untreated pregnant women with HIV/AIDS. BMJ Case Rep. 2020;13(3):e233426. doi:10.1136/bcr-2019-233426

The study patient, in early forties, in her fifth pregnancy with 25.3 weeks, was admitted with 2 months of dyspnoea. The patient had the following vital signs and findings on admission, normal heart rate and blood pressure, desaturation of 79% with FiO2 at 21%, evidence of left lung-basal crepitus, a gravid uterus and a normal fetal heart rate. Subsequently, the patient developed ventilatory failure needing invasive mechanical ventilatory support, with subsequent distributive shock requiring vasopressor and inotropic treatment. CT chest showed findings of a multilobar consolidative process with areas of frosted glass.

10.2 - Kherani RB, Shojania K. Septic arthritis in patients with pre-existing inflammatory arthritis. CMAJ. 2007;176(11):1605-1608. doi:10.1503/cmaj.050258

A 56-year-old woman presents to her family physician with a 7-day history of tenderness, swelling and redness of 2 metacarpophalangeal joints and 1 proximal interphalangeal joint in her right hand. She also reports pain and redness of her right knee that makes it difficult to walk. On examination, her right knee appears swollen and red. The patient has a 10-year history of rheumatoid arthritis with intermittent polyarticular flares, primarily involving her wrists, fingers (metacarpophalangeal and proximal interphalangeal joints), feet (metatarsophalangeal joints) and occasionally her knees. However, the condition has been well managed with methotrexate and hydroxychloroquine therapy. She had no flares in the year before presentation and is able to perform household chores and to work as a dry-cleaning attendant

Additional medical history was obtained, and the patient was examined fully. The patient denied factors that might predispose her to septic arthritis, including intravenous drug use, or adjacent skin trauma or infection. She also denied having other sites of infection or skin ulceration. The patient was not diabetic, nor did she have any prosthetic joints, urinary catheters or recent surgeries. She was not taking glucocorticoids or biologic agents. Apart from her musculoskeletal symptoms, she had felt vaguely unwell the week before presentation and felt that she was getting “worse.” The patient reported having intermittent night sweats. On presentation she was afebrile and normotensive but had tachycardia (102 beats/min). Findings on physical examination, including examination of the cardiopulmonary and dermatologic systems, were unremarkable.

14.2 - Fukuda H, Kondo Y, Nishina S, et al. A case of successful treatment with antiretroviral therapy for HIV in a patient with marked liver dysfunction. Heliyon. 2022;8(11):e11550. doi:10.1016/j.heliyon.2022.e11550

An otherwise healthy 67-year-old woman presented to a private clinic with common cold-like symptoms, and the clinician diagnosed her with upper bronchitis. She was prescribed garenoxacin mesylate hydrate, dimemorfan phosphate, and L-carbocysteine.

After 10 days from the appearance of the bronchitis symptoms, she was also presented to another hospital with severe fatigue, fever, and jaundice. Her initial findings were as follows: total bilirubin (T-Bil) 8.1 mg/dL, direct bilirubin (D-Bil) 5.8 mg/dL, alanine aminotransferase (ALT) 222 U/L, aspartate aminotransferase (AST) 115 U/L, prothrombin time (PT) 104%, activated partial thromboplastin time (APTT) 29.2 s. The patient was not a drug user or a commercial sex worker.

Initial laboratory values in our hospital and other liver biomarkers were as follows: T-Bil 10.2 mg/dL, ALT 166 U/L, AST 94 UL, INR 1.64, APTT 27.6 s.

Ultrasonography (US), computer tomography (CT), magnetic resonance image (MRI) and magnetic resonance cholangio pancreatography (MRCP) were initially considered to be performed as non-invasive examination methods. However, no stenosis was observed in the US or MRCP of the patient. Abdominal US at our hospital revealed a blunted rim of the liver and no dilation of the intrahepatic and extrahepatic bile ducts.

T2-weighted (T2W) MRI of the abdomen showed a high-intensity region around the intrahepatic portal vein, indicating abnormal periportal intensity.

Based on the provided information above, what are the top three most likely differential diagnoses in order from most to least likely?

15.2 - Ariff KM, Hassan ZA. Insomnia: Case Studies In Family Practice. Malays Fam Physician. 2006;1(1):11-14.

Mr. Tan, a 45-year-old man presents with difficulty falling asleep and staying asleep. The problem started after the death of his sister 2 months previously. He is unable to fall asleep until at least an hour after going to bed. He has no previous sleep problems. A general practitioner (GP) he consulted had prescribed low-dose dothiepin (tricyclic antidepressant) as sedation but Mr. Tan was unable to tolerate the drowsiness and dry mouth caused by the medication. He consumes 4 cups of coffee during the day and lately takes alcohol at night to aid sleep. The patient’s wife has noted that his legs jerk occasionally during the sleep though Mr. Tan is not aware of these movements.

Based on the provided information above, what are the top three most likely differential diagnoses in order from most to least likely?

16.2 - Kojima G, Tatsuno BK, Inaba M, Velligas S, Masaki K, Liow KK. Creutzfeldt-Jakob disease: a case report and differential diagnoses. Hawaii J Med Public Health. 2013;72(4):136-139.

The patient was a 66-year-old woman who was referred to a memory clinic for further evaluation of a 5-month history of rapidly progressive dementia. The initial symptoms included memory loss, “feeling odd,” anorexia, and unintentional weight loss. At her first visit to the memory clinic, her son and husband reported that her cognitive problems had acutely worsened in the previous two weeks. She now had problems with short-term memory and functional abilities, including getting dressed, using the toilet, and getting lost in her house. Her husband also stated that she had emotional lability and at times, did not trust her own family. Her vision was becoming blurry and she had increasing somnolence. Her family denied that she had any myoclonic jerks, tremor, gait unsteadiness, or visual, auditory, or sensory hallucinations.

Her past medical history was non-contributory. She had not had any previous surgeries, and her family history was significant for a myocardial infarction in her father and fatal leukemia in her mother, but there was no family history of dementia or prion disease. She had no known allergies and her medications consisted of naturopathic remedies that she started after the onset of her symptoms. She had travelled to Holland and Belgium 5 years previously, but was a strict vegetarian at that time. She had also travelled to the Caribbean, Mexico, and Belize, but did not have any exposure to raw livestock or brain matter. She had previously been a journalist in Washington DC, and did public relations for a real estate company in Hawai‘i.

Her physical exam was significant for perseveration, anomic aphasia, alexia, agnosia, and apraxia. Her muscle tone was normal in all four extremities and her cranial nerves were grossly normal. She had normal sensation and normal coordination. Her reflexes were symmetric and there were no Babinski reflexes. Her gait was slow but non-ataxic. She was unable to complete the Mini-Mental State Examination (MMSE) or perform other complicated tasks due to perseveration. For example, when asked about the month, date, day, and year, she answered “December” for each, when in fact, it was already March. When she answered “December” for the state, the MMSE was stopped. Blood work was normal and included a basic metabolic profile, CBC, thyroid studies, liver studies, vitamin B1, vitamin B12 and folate levels, lactic acid, erythrocyte sedimentation rate (ESR), rapid plasma reagin (RPR), human immunodeficiency virus (HIV), angiotensin-converting enzyme (ACE) levels, and ceruloplasmin. Pyruvate kinase, Purkinje cell antibody screen, Anti-Hu antibody, Anti-Jo antibody serum tests were also normal.

Cerebrospinal fluid (CSF) studies were also performed. Cell counts, glucose, and protein were within normal limits. Toxoplasma gondii, Bartonella DNA, venereal disease research laboratory (VDRL) test, and Lyme antibodies were negative. Her 14-3-3 protein level was also within normal limits at less than 1.0 ng/mL, with a normal reference range of less than 1.5 ng/mL.

A non-contrast Magnetic Resonance Imaging (MRI) of her brain was significant for global parenchymal loss. Diffusion-weighted images showed restricted cortical diffusion in the cingulate gyrus (Figure 1) and also in the bilateral parietal and posterior temporal lobes (Figure 2). Mildly restricted diffusion was seen in the thalami (Figure 3). There were no infarcts, masses, or extra axial fluid collections. An electroencephalogram (EEG) showed left temporal slowing with a diffusely slow and disorganized background, and there were no periodic discharges noted.

18.2 - Park HJ, Kang MI, Kang Y, et al. Two cases of refractory thrombocytopenia in systemic lupus erythematosus that responded to intravenous low-dose cyclophosphamide. J Korean Med Sci. 2013;28(3):472-475. doi:10.3346/jkms.2013.28.3.472

A 47-yr-old woman was admitted for 2-month recurrent oral ulcer, anemia, and skin lesions, including purpura and petechiae, on August 14, 2010. On admission, laboratory results showed WBC and platelet counts of 3,700/µL (neutrophils, 55.6%; lymphocytes, 34.5%) and 5,000/µL, respectively. The hemoglobin concentration was 7.4 g/dL; the corrected reticulocyte percentage was 1.15%; the ESR was 33 mm/hr; and the CRP concentration was < 0.1 mg/L. Levels of ferritin and LDH were not elevated, to 4.2 ng/mL (normal: 11-306 ng/mL) and 223 IU/L (normal: 119-247 IU/L), respectively. Neither proteinuria nor hematuria was observed. ANA was detected at a titer of 1:640, but anti-dsDNA was negative. Complements 3 and 4 were 78.7 and 11.5 mg/dL, respectively.

Abdominal ultrasonography showed no splenomegaly. And a bone marrow biopsy showed 50%-60% cellularity and abundant megakaryocytes.

She received intravenous dexamethasone (20 mg/day) and immunoglobulin (5 g/day) for 5 days, and then was maintained on a moderate dose of prednisolone (30 mg/day). However, 1 month later, her platelet count had decreased to 9,000/µL. She received pulsed intravenous methylprednisolone (1,000 mg/day) for 3 days and immunoglobulin (5 g/day) for 5 days, followed by oral prednisolone (1 mg/kg/day). However, this combination treatment showed no efficacy. She started low-dose IV CYC (500 mg/day, biweekly) on September 16 and achieved induction of remission after three cycles of CYC. After she finished six cycles of low-dose IV CYC therapy in the outpatient clinic, started AZA (50 mg/day) with reducing the dose of prednisolone to 15 mg/day. However, she switched from AZA to MMF (500 mg/day) because her platelet count was decreased to 41,000/µL when she was maintained on AZA. After maintenance on MMF, she showed a stable platelet count without relapse into thrombocytopenia or complications due to the medication after 24 months.

Based on the provided information above, what are the top three most likely differential diagnoses in order from most to least likely?

19.2 - Fischer Q, Brillat-Savarin N, Ducrocq G, Ou P. Case report of an isolated myocarditis due to COVID-19 infection in a paediatric patient. Arzanauskaite M, Voges I, Tirr E, Fielder Camm C, Thomson R, eds. European Heart Journal - Case Reports. 2020;4(FI1):1-5. doi:10.1093/ehjcr/ytaa180

A 15-year-old boy without cardiovascular risk factors or previous history of cardiovascular disease presented to the emergency department in our institution for persistent chest pain with mild fever (<38°C) for the last 3 days. The patient reported no respiratory tract signs.

The physical examination revealed blood pressure of 100/60 mmHg, heart rate of 75 b.p.m., oxygen saturation of 98% while breathing ambient air, and body temperature of 36.9°C. The electrocardiogram showed diffuse ST elevation without reciprocal changes

Blood tests revealed a slight increase in C-reactive protein level (41 mg/L, normal <6 mg/L) with normal leucocytes (6.1 × 109/L, normal 4–10 × 109 cells/L) and elevated cardiac troponin 6.1 μg/L (99th upper reference limit 0.045 μg/L). N-terminal probrain natriuretic peptide (NT-proBNP 65 ng/L, normal <300 ng/L) and D-dimer (259 ng/mL, normal <500 ng/mL) remained normal.

Because of systematic suspicion of COVID-19 in patients with unexplained fever, a PCR was performed on a nasopharyngeal swab and resulted positive for SARS-CoV-2. A multiplex real-time PCR was also performed and resulted negative, allowing the exclusion of viral co-infections. Chest CT scan showed no lung anomalies (Supplementary material online). There were no other family members tested positive for SARS-coV-2.

20.2 - Chang PH, Jeng CM, Chen DF, Lin LH. A case report and literature review of sigmoid volvulus in children. Medicine (Baltimore). 2017;96(52):e9434. doi:10.1097/MD.0000000000009434

This 12-year-old boy presented with autism and a history of hearing impairment in his right ear. He had been well until about 1 week before this presentation, when nausea, persistent abdominal distention, poor appetite, and reduced activity were noted by his parents. He did not have a fever or diarrhea. His family brought him to the Emergency Department of Cathay General Hospital on September 21, 2016. A physical examination at admission revealed a massively distended abdomen without muscle guarding or rebounding pain. The initial laboratory tests showed a white blood cell count of 9.88 × 103 cells/mm3 [normal reference (NR): 4–10 × 103/μL] with elevated segments (86.4%) [NR: 40–75%], normocytic anemia (Hb: 13.9 g/dL; MCV: 82 fL) [NR: Hb: 14–18 g/dL; MCV: 81–97 fL] and a C-reactive protein (CRP) level of 0.284 mg/dL [NR: 0.01–0.5 mg/dL]. Abdominal plain film revealed severe colonic distention with gas over his abdomen suggesting ileus (Fig. ​(Fig.1).1). He was then admitted under the tentative diagnosis of abdominal distention with unknown cause, and intravenous metoclopramide was initially given empirically.

To rule out acute gastroenteritis or infectious colitis, we tested for rotavirus antigen which showed negative results, and a stool culture/analysis revealed no significant findings with no parasite ova or occult blood. Hirschsprung disease (HD) was actually not in our consideration. According to our patient's history, he did not fail to pass the meconium within 48 hours of delivery which is typical HD symptom. There were no vomiting green or brown substance, bloody diarrhea, swollen belly, excessive intestinal gas, and explosive stools after a doctor inserts a finger into the rectum before his age of 10. Therefore, HD could be excluded (Fig. ​(Fig.22).

23.2 - He J, Li J, Li Z, Ren H, Chen X, Tang J. A Case Report of Wernicke’s Encephalopathy Associated With Schizophrenia. Front Psychiatry. 2021;12:657649. doi:10.3389/fpsyt.2021.657649

A 56-year-old female patient with a pertinent history of schizophrenia, primary angle-closure glaucoma with the left eye, chronic bronchitis, coronary heart disease, and renal calculi was brought to the emergency department with altered mental status and mild respiratory disorder. Three months ago, after drug withdrawal without the guidance of doctors, she began to have near-total absence of motion and speech and refused to take antipsychotics and food. Even though she had been hospitalized in another hospital a month earlier and given antipsychotics and nutritional support, her condition had not improved. A day earlier, the patient had been noted to open her mouth to breath, followed by the whirring sound. According to her husband, she had no history of alcohol, tobacco, or substance abuse. However, her compliance with antipsychotics was so poor that the control of psychosis was relatively ineffective, and her ability to contact and communicate had been impaired in recent years. Neither she nor her family could provide further details.

Her physical examination revealed that she had a weight of 45 kg, a temperature of 36.4C, a blood pressure of 129/84 mmHg, and a pulse of 105/min. She was bed-bound, except for relieving herself, clear in consciousness with a blank and blunted facial expression, and refused to answer questions. She was unable to cooperate with most of the physical examinations because she refused to follow instructions. Her bilateral pupils were equal and round. The left pupil was fixed and had no light reflex and the right pupil was reactive to light. Of note is that her left horizontal gaze palsy was obvious, but her eyes were closed tightly most of the time. She presented with increased muscular tension and tendon hyperreflexia in extremity. Other than that, the remainder of the neurologic examination was unremarkable or uncompleted.

Her initial laboratory workup was as follows: white blood cell count, 13.03 × 103/μL with 88.80% neutrophils; C-reactive protein, 21.2 mg/L; procalcitonin, 0.31 ng/L; myoglobin, 81 μg/L; and ischemia-modified albumin, 57.6 μ/ml; electrolytes, the level of thyroid hormones, and liver and renal function tests were otherwise unremarkable. Her plasma lactate level was 2.24 mmol/L, which increased very slightly. Chest CT showed diffuse infiltration in her right middle lobe and inferior lobes of bilateral lungs. Her electrocardiogram and brain CT were normal. The respiratory consultant diagnosed her with aspiration pneumonia caused by prolonged bed rest and improper breathing pattern.

She was admitted to the psychiatry department at first, with nasogastric feeding. No antipsychotics were applied because of the diagnosis of malignant syndrome that needs to be ruled out. After being given oral and parenteral nutrition for 3 days, she seemed to get better. She removed the nasogastric tube by herself for feeling uncomfortable and ate a little porridge. She could complete some simple instructions like “drink water” and the whirring sound associated with breath became less. While on hospital day 5, the patient suddenly had difficulty in breathing and became confused. Arterial blood gas demonstrated type I respiratory failure with a partial pressure of carbon dioxide of 41.60 mmHg and a partial pressure of oxygen of 53.60 mmHg. Saturation of blood oxygen fluctuated between 63 and 83%, increasing immediately after airway suction. The respiratory consultant explained that the phenomenon was a result of a massive airway secretion produced through mouth breathing.

On hospital day 6, the patient was eventually transferred to the neurology intensive care unit and a lumbar puncture and a brain MRI were performed. Cerebrospinal fluid (CSF) biomarkers were normal, except for a slightly elevated glucose (5.51 mmol/L). The brain MRI showed symmetrical increased T2 signal and restricted diffusion in the bilateral basal ganglia and around the third ventricle

78.2 - Sheibani H, Javedani Masroor M. Pericarditis as a trigger for Prinzmetal angina - a case report. J Med Life. 2021;14(6):853-861. doi:10.25122/jml-2021-0061

The patient was a 28 years old man, an ordinary worker without any remarkable history and cardiovascular risk factors, who was admitted with a complaint of retrosternal chest pain and heaviness one hour before hospitalization in the emergency room. The first electrocardiogram demonstrated localized ST-segment elevation with a pattern in favor of acute infero postero-right ventricular (RV) myocardial infarction, accompanied by generalized ST-segment elevation in precordial leads in favor of acute pericarditis (Figures 1, 2).

After verifying the patient’s medical history, we identified that the patient was hospitalized two months ago, with acute pericarditis, generalized ST-elevation, and normal transthoracic echocardiography, and received treatment for acute pericarditis. Following a good response to treatment, the patient was discharged in good general condition with ibuprofen (Figure 3).

The patient continued to take ibuprofen for 10 days after discharge. Twenty-four hours prior to this hospitalization, he suffered from pleuritic chest pain similar to the pain of the previous hospitalization and did not take any medication. However, one hour before hospitalization, he suffered from severe retrosternal pain with nausea, sweating, shortness of breath, lethargy, and mild weakness, which increased in severity. The patient was alert during the physical exam with stable vital signs and mild cold sweating, blood pressure (BP)=110/70, pulse rate (PR)=60 beats per minute (BPM), respiratory rate (RR)=15/M, oxygen saturation level 98%; other physical exams were unremarkable.

According to dual type, ECG changes as the patient received ASA 300 mg, Plavix 300 mg, Pearl TNG, and Atorvastatin 80 mg in the emergency room. Transthoracic echocardiography revealed that the inferior wall had mild hyperkinesia and mild right ventricle systolic dysfunction.

The patient was transferred to CCU with a diagnosis of infero postero-RV MI. In CCU, the patient received anti-ischemic therapy, including Trinitroglycerin (TNG) infusion with the minimum dose and recurrent BP check. Retrosternal chest pain relatively subsided and returned ST-elevation near baseline in II, III, AFV, RV4 (Figure 4).

Due to good blood pressure, low age, and good performance of the right ventricle (RV) in TTE, TNG infusion continued. The first qualified troponin I was negative. 20 minutes later, ECG returned all ischemic changes to baseline, but generalized ST-elevation remained in favor of pericarditis (Figure 5).

At the same time, the patient’s retrosternal pain completely disappeared and the pleuritic pain, although not eliminated, decreased in severity. The patient’s second and third troponin was also negative.

Creatine phosphokinase (CPK), creatine phosphokinase myocardial band (CPK MB), C-Reactive protein (CRP), erythrocyte sedimentation rate (ESR) were performed, all of which were in the normal range. No arrhythmias or recurrent pain recurred during the four-day hospitalization (Figure 6).

The complete disappearance of ischemic symptoms and partial reversal of pericarditis changes on ECG are evident. On the third day, he underwent coronary angiography, which revealed that the epicardial arteries were open and not narrow and without stenosis (Figure 7).

Transthoracic echocardiography (TTE) demonstrated normal ejection fraction without structural heart abnormality. He was discharged in good general condition on the fourth day and treated with ibuprofen, colchicine, and diltiazem. Two weeks later, all ECG changes returned, and ECG was near normal, with no new arrhythmias or changes (Figure 8).

Our patient had no new problems within three months after recovery and treatment with a calcium blocker during the next follow-up.

83.2 - Unruh A, Fitze G, Jänig U, Bielack S, Lochbühler H, Coerdt W. Medullary thyroid carcinoma in a 2-month-old male with multiple endocrine neoplasia 2B and symptoms of pseudo-Hirschsprung disease: a case report. Journal of Pediatric Surgery. 2007;42(9):1623-1626. doi:10.1016/j.jpedsurg.2007.05.015

The patient was delivered as the second twin of a second gravida by cesarean section in the 37th week of an uncomplicated pregnancy. No abnormalities of facies or habitus were detected. After an uneventful perinatal period, the patient was admitted at 9 days of life because of weight loss, failure to thrive, and hyperbilirubinemia. No septic or metabolic disorders were found. Supplementary feeding by nasogastric tube was necessary for adequate food intake. At the age of 1 month, the patient was referred to our hospital with a putative diagnosis of Hirschsprung disease, with progressive abdominal distension and explosive bowel movements several times a day, yet no vomiting. The patient was still tube-fed.

An abdominal radiograph showed distended small and large bowel loops consistent with a distal passage obstruction, whereas the colon enema showed a funnel-like rectosigmoid transition zone and a barely filled rectum. Rectal biopsies were taken from the mucosa at 1, 2, 6, and 7 cm ab ano. Histochemical examination in all specimens within the muscularis mucosae showed a negative acetylcholinesterase (AChE) reaction. Despite aganglionosis being the typical sign for Hirschsprung disease, ganglioneuromatous hypertrophy of the submucosal plexus was seen in all specimens (Fig. 1), which warranted further diagnostics, as follows.

The molecular genetic analysis of the RET protooncogene revealed a heterozygous M918T mutation on exon 16. The serum calcitonin level was markedly elevated at 1150.9 pg/mL (normal, <8.4 pg/mL). Except for an irregular area of tissue of less than 3 mm in the left lobe, imaging of the thyroid gland by ultrasound and magnetic resonance imaging (MRI) did not show any tumor-like alteration. No pathological uptake of contrast medium or suspicious lymph nodes was seen.

Total thyroidectomy with excision of 3 central lymph nodes was performed at 9 weeks of age. Histology of the excised thyroid gland revealed a distinct nodular C-cell hyperplasia in both lobes as well as a cord-like infiltration of tumor cells beyond the follicles, compatible with a medullary microcarcinoma (Fig. 2).

Two months after the operation, the basal serum calcitonin levels were still elevated with 14.1 pg/mL, increasing up to 22.7 pg/mL after pentagastrin stimulation. No suspicious signs of lymph node or distant metastases were seen in either ultrasound, computed tomography of the chest, MRI scans of neck and thorax, or in a 99mTc bone scintigraphy. When serum calcitonin levels increased during follow-up to 18.7 pg/mL, a bilateral neck dissection was carried out 9 months after the thyroidectomy. Thirty-nine excised lymph nodes were histologically free of metastases. Postoperatively, serum calcitonin was still elevated at 31.1 pg/mL. Repeated postoperative ultrasound scans, an MRI scan of the neck and chest, and a somatostatin receptor scintigraphy did not reveal any intrathoracic masses or nodes. No evidence for the presence of ectopic thyroid tissue could be found.

Since the first operation, follow-ups have included the control of serum levels of calcitonin, thyroxin, thyroid stimulating hormone (TSH), calcium, parathyroid hormone, and catecholamine-metabolites in the urine. Thyroxin has been substituted in weight-adapted doses, and calcium has been administered because of a hypoparathyroidism.

From the standpoint of the gastrointestinal symptoms, the patient improved clinically with regular rectal enemas, which were started at 5 weeks of age. Tube feeding has not been further necessary, and the patient thrives currently well, yet daily enemas are still used for defecation and the abdomen remains distended, suggesting a chronic constipation. However, there have been no episodes of abdominal pain or vomiting, and thus no surgical therapy for the constipation has been far recommended.

Molecular genetic analysis of the 2 siblings and of both parents—all clinically healthy—showed normal chromosomal constellations and no mutations of the RET protooncogene (Fig. 3). A genetic counseling of the family has been carried out because of the 50% chance of inheritance of the MEN 2B syndrome in the patient's offspring.

84.2 - Gammon B, Robson A, Deonizio J, Arkin L, Guitart J. CD8+ granulomatous cutaneous T-cell lymphoma: A potential association with immunodeficiency. Journal of the American Academy of Dermatology. 2014;71(3):555-560. doi:10.1016/j.jaad.2014.03.028

A 49-year-old man with common variable immuno-deficiency (CVID) presented with a 1-year history of progressive, asymptomatic red papules and nodules on his trunk and extremities. He

had been previously diagnosed with cutaneous T-cell lymphoma (CTCL), not otherwise specified (NOS) at an outside facility and began a course of oral bexarotene up to a dose of 300 mg daily without response. He subsequently also developed destructive arthritis of the bilateral metacarpophalangeal joints and was diagnosed with rheumatoid arthritis for which he was prescribed hydroxychloroquine, methotrexate, and prednisone with some relief of symptoms. The physical examination revealed indurated, erythematous, dome-shaped papules, nodules, and plaques without surface change or necrosis on the upper and lower extremities, scalp, and both the left and right sides of the abdomen (Fig 1, A and B). Lymphadenopathy was not identified. The patient’s laboratory and imaging findings were remarkable for a baseline undetectable immunoglobulin A (IgA) and IgM, and a fluorode-oxyglucoseepositron emission tomography (FDG-PET) scan revealed patchy nodular and ground glass opacities with moderate FDG avidity in both lungs, as well as small scattered cutaneous foci of FDG avidity. The patient’s complete blood cell count (CBC), complete metabolic panel (CMP), viral hepatitis panel, serum angiotensin-converting enzyme level, serum and urine protein electrophoresis, extractable nuclear antigen profile, and complement levels were all within normal limits. A biopsy specimen of a prototypical lesion was obtained, and it revealed a multinodular pandermal infiltrate (Fig 2, A) comprised of epithelioid histiocytes with occasional multinucleated giant cells admixed with a dense lymphocytic infiltrate (Fig 2, B) comprised of intermediate, elongated atypical cells (Fig 2, C ) extending into the reticular dermis. Angiocentricity or angiodestruction were not appreciated. The infiltrate was composed of primarily of CD31CD81 T cells (Fig 2, D) that expressed the cytotoxic markers T cellerestricted intracellular antigen 1 (TIA-1; Fig 2, E ) and granzyme B (Fig 2, F ). Special stains (ie, periodic acideSchiff, diastase resistant [DPAS],

Gram, and acid-fast bacilli) were negative for microorganisms. In situ hybridization for Epstein Barr virus (EBV) was negative. A clonal T cell population was detected in the skin using a

polymerase chain reaction (PCR) study. The patient began a course of gemcitabine with

some initial improvement in his skin disease, followed by relapse and disease progression.

85.2 - Honeybul S, Lang DA, Howard D. Group B streptococcal cervical osteomyelitis in a neonate. Journal of Clinical Neuroscience. 2006;13(5):607-612. doi:10.1016/j.jocn.2005.07.010

A 4-week-old boy was admitted to a peripheral hospital following an episode of being pale, grey and floppy. His mother had also heard ‘crunching noises’ while he breast-fed, and he had seemed to be distressed when moving his neck. He was one of a set of twins born at 37 weeks via an uncomplicated emergency caesarean section. His sister remained well. On initial examination he was found to be pyrexial, but had no neurological deficits. Following a presumptive diagnosis of late onset GBS septicaemia, antibiotic treatment was commenced. After an initial improvement over 4 days, he was noted to be increasingly distressed when moving his neck and became episodically hypotonic. A neck radiograph confirmed vertebral collapse of C3 (Fig. 1), and he was transferred to the neurosurgical unit for further investigation.

On initial clinical examination the patient was found to have increased upper and lower limb tone, and spontaneous movements of the lower limbs and right upper limb, but minimal movement of the left hand in response to painful stimuli. He held his head rotated to the right. Haematological analysis confirmed a C-reactive protein level of 77 mg/L and a white cell count of 45 × 109/L. A computed tomography (CT) scan of the cervical spine confirmed destruction of the body of C3. Initial attempts at magnetic resonance imaging (MRI) were unsuccessful due to movement artefact.

Following a presumptive diagnosis of vertebral osteomyelitis, the patient was nursed in a neutral position with his neck supported with a soft collar and tape. Cefotaxime and rifampicin treatment was commenced. Initially, the patient made satisfactory progress: limb movement improved, and his inflammatory markers returned to normal levels. However, one week following admission his condition deteriorated. He became tachypnoeic, hypoxic, clammy and developed apnoeic episodes. Movements of both upper limbs decreased.

Given the poor quality of the initial MRI and his clinical deterioration, it was felt that more accurate soft tissue imaging was required. The patient was transferred to the paediatric intensive care unit, where he was intubated and ventilated. He was then transferred for MRI scanning on a spinal board. The MRI confirmed not only destruction of C3 but also C2 and C4, with kyphotic angulation and anterolisthesis at C3/4 (Fig. 2). In addition, there was enhancing soft tissue around the cord and abnormal signal within the cord.

86.2 - Brikman S, Denysova V, Menzal H, Dori G. Acute pancreatitis in a 61-year-old man with COVID-19. CMAJ. 2020;192(30):E858-E859. doi:10.1503/cmaj.201029

On day 14, our patient reported sudden diffuse abdominal pain with anorexia. He was afebrile and had no vomiting or diarrhea. His abdomen was soft, with mild diffuse tenderness but no signs of peritoneal irritation. A complete blood cell count showed his level of leukocytes was elevated at 37 (normal 4.5–11.5) × 109/L with 93% neutrophils, and he had an absolute lymphocyte count of 0.98 (normal 1.5–5.0) × 109/L. His serum lipase level was elevated at 203 (normal 21–67) U/L, and he had a serum amylase level of 142 U/L (28–100 U/L). His levels of alkaline phosphatase and direct bilirubin were in the normal range. Contrast-enhanced computed tomography (CT) of our patient’s abdomen showed signs of pancreatitis

88.2 - Arnold FW, Mahmood K, Prabhu A, et al. COPD exacerbation caused by SARS-CoV-2: A Case Report from the Louisville COVID-19 Surveillance Program. The University of Louisville Journal of Respiratory Infections. 2020;4(1). doi:10.55504/2473-2869.1092

A 53-year-old male with underlying chronic obstructive pulmonary disease (COPD) and chronic respiratory failure on home oxygen per nasal cannula during the day and non-invasive positive pressure ventilation (NIPPV) at night was referred to the emergency department (ED) from the pulmonology clinic for worsening dyspnea and hypoxia. The patient was recently treated as an outpatient with oral azithromycin and prednisone for a suspected COPD exacerbation five days prior to admission. The patient had symptoms of purulent phlegm with an oxygen saturation in the 70s at home. In the ED, the patient had a temperature of 97.7°F with a prolonged expiratory phase of breathing and wheezing as well as diminished breath sounds. He was noted to have acute on chronic hypoxic/hypercarbic respiratory failure and was admitted to the ICU for further management. His arterial blood gas showed a pH of 7.27, a partial pressure of carbon dioxide (pCO2 ) of 73, a partial pressure of oxygen (pO2 ) of 185, a bicarbonate of 32.6, a base excess of 3.2 on 60% fraction of inspired oxygen (FiO2 ) revealing respiratory acidosis. A rapid influenza screen was negative. His chest x-ray revealed COPD changes and no acute infiltrate (Figure 1). After admission to the ICU, the patient was placed on NIPPV with improvement. Azithromycin and steroids were again initiated for an acute exacerbation of COPD. The patient was also given oseltamivir due to a history of exposure to a contact with influenza virus. He was stabilized in the ICU and was subsequently transferred to the medical floor within 24 hours of hospitalization. A respiratory viral/bacterial panel was negative.

89.2 - Khan TV, Toms C. Cytomegalovirus Colitis and Subsequent New Diagnosis of Inflammatory Bowel Disease in an Immunocompetent Host: A Case Study and Literature Review. Am J Case Rep. 2016;17:538-543. doi:10.12659/ajcr.898005

A 40-year-old man with no relevant past medical history presented to the ED with a one-week history of diarrhea with fevers to 38.7 degrees Celsius, bright red blood per rectum, light headedness, and dizziness for the past eleven days. Patient had a similar episode about seven months prior which resolved spontaneously after three weeks. He denies recent foreign travel or exposure to sick contacts.

On admission his physical examination was benign other than diffuse abdominal tenderness, mild jaundice, and blood on digital rectal exam. Labs revealed a transaminitis but negative viral hepatitis (Table 1). Computed tomography of the abdomen with oral and intravenous contrast showed nonspecific colitis.

He underwent a flexible sigmoidoscopy which showed diffuse inflammation and crypts along with gross blood. Given the clinical picture along with the findings on flexible sigmoidoscopy, CMV was suspected and pathology samples sent for testing. Once they returned positive, he was started on IV Ganciclovir.

Pathological samples from flexible sigmoidoscopy and colonoscopy demonstrated acute cryptitis with crypt abscess formation, characteristic of acute colitis. Some of the crypts were branched and others absent, evincing a previous bout of destruction and regeneration (crypt-architectural distortion). This finding is characteristic of idiopathic inflammatory bowel disease. Closer inspection of the lamina propria demonstrated the presence of characteristic intranuclear (“owl’s eye”) and intracytoplasmic (tiny eosinophillic globules) CMV inclusions. The presence of CMV inclusions was confirmed immunohistochemically (Figures 1​1–3).

Over the next few days the patient began to have a decrease in the frequency of bowel movements, remained afebrile, and was able to tolerate oral intake without nausea along with a positive trend in his labs, as seen in Table 1, and was thus discharged home. One month after discharge, a colonoscopy to assess bowel pathology demonstrated ongoing colitis from the rectum to the hepatic flexure and a normal appearing terminal ileum and ascending colon. Biopsies were consistent with Inflammatory Bowel Disease (Figure 4).

92.2 - Goh BKP, Tan Y, Chang KTE, Eng PHK, Yip SKH, Cheng CWS. Primary Hyperaldosteronism Secondary to Unilateral Adrenal Hyperplasia: an Unusual Cause of Surgically Correctable Hypertension. A review of 30 cases. World j surg. 2007;31(1):72-79. doi:10.1007/s00268-005-0594-8

A 56-year-old man was newly diagnosed with hypertension 2 months prior to admission with hypokalemic periodic paralysis. On admission, he had a poorly controlled blood pressure of 180/100 mmHg, a low serum potassium concentration of 1.6 mmol/l, normal sodium of 138 mmol/l, and a serum bicarbonate of 31.2 mmol/l. His thyroid function test was normal. After correction of his hypertension and hypokalemia, he was discharged with long-acting nifedipine [30 mg every morning (o.m.)], atenolol (50 mg o.m.), and potassium supplement of 1.2 g o.m. He was followed up at the outpatient clinic and had a blood pressure of 160/100 mmHg and potassium of 3.5 mmol/l on the above medications. Biochemical evaluation revealed a random plasma aldosterone of 642 pg/ml and plasma renin activity (PRA) of 0.15 ng/ml per hour. The 24-hour urine collection showed a urinary aldosterone of 37.3 μg/day (normal: 6.0–25 μg/day) and a urinary potassium of 93 mmol/day (normal: 4–42 mmol/day). He subsequently underwent a postural test after salt loading. At 08:00 in a supine position, the PRA was 0.22 ng/ml per hour, plasma aldosterone was 113 pg/ml, and cortisol was 361 nmol/l. At 12:00, in an erect position, his PRA was not detectable, plasma aldosterone was 129 pg/ml, and cortisol was 138 nmol/l. The 24-hour urine collection showed urine aldosterone 37.8 μg/day and urine potassium of 85 mmol/day. A computed tomography (CT) scan was then performed, which revealed a 1-cm nodule in his right adrenal gland, suggesting an adrenal adenoma and a normal left adrenal gland. He subsequently underwent laparoscopic right adrenalectomy, which was uncomplicated. Histological examination of the gland showed nodular hyperplasia with no adenoma. There were several uncapsulated circumscribed nodules composed of nests of cells with abundant pale cytoplasm (Fig. 1). He was followed up for 20 months and remained normotensive without antihypertensives and had a normal serum potassium concentration.

93.2 - Roje Z, Roje Ž, Matić D, Librenjak D, Dokuzović S, Varvodić J. Necrotizing fasciitis: literature review of contemporary strategies for diagnosing and management with three case reports: torso, abdominal wall, upper and lower limbs. World J Emerg Surg. 2011;6(1):46. doi:10.1186/1749-7922-6-46

A 63 years old, paraplegic and diabetic (type I) male patient was admitted to the Emergency department because of a two week history of high fever, perirectal pain, purulent drainage and a clinical picture of bacterial sepsis (Table 1). His diabetes mellitus was treated with insulin injections. He had pressure sores on the greater trochanter of right leg and sacral region which were treated with serial debridements and drainages on an outpatient basis by his family doctor during the previous two months. In his acute clinical status we found perianal induration with perianal abscesses and large grade III/IV sacral and trochanteric pressure sores, with multiple drainage sinuses. In both inguinal regions the patient had erythema and crepitations, stronger on the left side. The scrotal skin region was painful, edematous, and pruritic. On the left knee region there was an additional pressure sore with edema, fluid collections and lymphangitis in the ipsilateral inguinal region. His laboratory blood values showed signs and symptoms of SIRS with hyperglycemia of 21 mmol/L, a total leukocyte count of 6.35 × 109 with 78% of PMNs, 17% bands, 11% lymphocytes, and 120 × 109/L platelets, hemoglobin of 78 g/L, albumin 29 g/L, protein 61 g/L CRP of 69,1 mg/L, and creatinin 216 mmol/L. The patient was febrile, with symptoms of systemic toxicity. In his local status he had scrotal gangrene, fulminating perineal abscesses and a fluid collection with crepitations on the left thigh. The plain film radiography of the pelvic region showed the presence of gas in the perineum. CT scan of the left thigh revealed suspected septic arthritis secondary to the pressure sore in the knee region, and low attenuation in vastus lateralis muscle, and gas in both perineal regions. The diagnosis of Fournier's gangrene was reached based on clinical examination and laboratory findings.

94.2 - Matos AM, Oliveira RRD, Lippi MM, Takatani RR, Oliveira Filho WD. Use of noninvasive ventilation in severe acute respiratory distress syndrome due to accidental chlorine inhalation: a case report. Revista Brasileira de Terapia Intensiva. 2017;29(1). doi:10.5935/0103-507X.20170015

A 55-year-old man accidentally inhaled a chlorine cloud when cleaning the swimming pool at his home, evolving to a clinical picture of mild dyspnea, cough with mucoid sputum, and epigastric pain. He sought emergency care (after 30 minutes), where he was initially evaluated for respiratory symptoms and received venous hydration, bronchodilators, and oxygen therapy. However, he presented progressive clinical worsening over a 3-hour period, with increased expectoration, arterial oxygen saturation (SpO2) (from 95% to 60%), and cyanosis and was referred to the reference emergency room, where he was seen in the emergency department. At that time, he already had signs of acute respiratory failure, associated with intense burning chest pain and cough with blood-tinged sputum. There was no history of smoking, respiratory diseases, and other comorbidities.

At admission, the patient was afebrile, tachycardic (heart rate 110bpm), and tachypneic (respiratory rate 34bpm), with a blood pressure of 134/82mmHg and an SpO2 of 86% during oxygen macronebulization at 10L/minute. Chest expandability was decreased due to pain, and respiratory auscultation detected the presence of generalized decreased vesicular murmur and crackling rales mainly on the lung bases. No other changes were detected on physical examination. Noninvasive ventilation with 60% FiO2, 7cmH2O support pressure (SP), and 10cmH2O positive end expiratory pressure (PEEP) was started.

95.2 - Eriksson C, Gustavsson A, Kronvall T, Tysk C. Hepatotoxicity by bosentan in a patient with portopulmonary hypertension: a case-report and review of the literature. J Gastrointestin Liver Dis. 2011;20(1):77-80.

The patient was a 29-year-old woman, who underwent splenectomy in 2001 due to idiopathic thrombocytopenic purpura unresponsive to corticosteroid therapy. In 2007 she suffered from increasing fatigue, severe dyspnoea and right-sided pleural effusion. A diagnosis of PPHTN was made after cardiovascular and hepatologic investigation. Pulmonary artery pressure was increased to 107/40 mmHg, mean 65 mmHg, and systolic function of the right ventricle was impaired. An abdominal CT scan revealed findings of a previous portal vein thrombosis with an occluded portal vein, multiple collaterals and oesophageal varices were diagnosed at gastroscopy. Liver function tests were normal apart from International Normalized Ratio (INR) 1.3 (ref value 0.9-1.1). A liver biopsy revealed only hepatic steatosis. No underlying myeloproliferative or coagulation disorder was found and the portal vein thrombosis was considered as postoperative after previous splenectomy. Treatment of pulmonary hypertension with furosemide, spironolactone and bosentan in increasing dose up to 125 mg twice daily was introduced. Oesophageal varices were treated with primary endoscopic ligation, as betablockers were considered inappropriate with respect to her cardiovascular disease due to their negative inotropic and chronotropic effects. The patient improved and liver function tests remained normal during regular follow-up.

96.2 - Hahn SJ, Park J hyun, Lee JH, Lee JK, Kim KA. Severe Hypertriglyceridemia in Diabetic Ketoacidosis Accompanied by Acute Pancreatitis: Case Report. J Korean Med Sci. 2010;25(9):1375. doi:10.3346/jkms.2010.25.9.1375

A 20-yr-old female visited the emergency department because of a 1-day history of vomiting (10 times) and was experiencing epigastric pain with diarrhea on March 23, 2009. The upper gastric pain was continuous without radiation. The patient had been drinking almost daily alcoholic beverages soju (alcohol concentration in the range of 19-22%) for 5 days prior to admission. The patient had a smoking history of one pack-year. Two years previously, the patient experienced DKA accompanied by acute pancreatitis. At that time, the patient had been diagnosed with type 1 diabetes mellitus. Insulin treatment began at that time. However, 7 months prior to the current admission, the patient ceased taking insulin.

Upon admission, the patient was determined to be 161 cm in height, 55 kg in weight with a body mass index of 21.2. On admission, the patient was alert but appeared acutely ill. Initial vital signs were blood pressure 90/60 mmHg, pulse rate of 88 beats/min, respiratory rate of 20/min and body temperature of 36.5℃. Physical examination revealed a dehydrated tongue and skin turgor. There was no evidence of xanthoma, xanthelasma or eruptive xanthoma. No palpable lymph node enlargement was apparent on head and neck examination, and no abdominal tenderness on abdominal examination. Bowel sound was normoactive.

Initial laboratory findings were ABGA (pH 7.148, pCO2 12.9 mmHg, pO2 126 mmHg, HCO3- 8.4 mM/L, SaO2 98.0%), glucose level 281 mg/dL, hemoglobin A1c 13.8% , C-peptide (premeal) 0.441 ng/mL (normal reference: 1.1-4.4 ng/mL), total cholesterol 1,640 mg/dL, TG 15,240 mg/dL, measured low density lipoprotein cholesterol (LDL-C) 246 mg/dL (determined by homogeneous enzymatic colorimetry method assay), high density lipoprotein cholesterol (HDL-C) 69 mg/dL, serum ketone body 3.1 mM/L (normal reference 0-0.05 mM/L), total bilirubin 1.2 mg/dL, AST 19 IU/L, ALT 14 IU/L, total protein 8.4 g/dL, serum albumin 4.1 g/dL, alkaline phosphatase 147 IU/L, serum amylase 81 U/L, serum lipase 108 U/L, WBC 13,310/µL, hemoglobin 13.9 g/dL, hsCRP 5.616 mg/dL, BUN 14.0 mg/dL, creatinine 0.6 mg/dL, sodium 125 mEq/L, potassium 4.4 mEq/L and chloride 95 mEq/L. There were no abnormalities in the coagulation test. Serum sample was milky and turbid, which suggested a lipemic state (Fig. 1). Anti-glutamic acid decarboxylase (GAD) antibody was 0.12 U/mL (normal reference 0-0.9 U/mL), and anti-islet antibody-2 (IA-2) antibody was <0.4 U/mL (normal reference 0-0.4 U/mL). Apolipoprotein E genotyping assessed by polymerase chain reaction revealed ε2/ε3.

The patient was aggressively hydrated and treated with intravenous insulin in the intensive care unit. On admission, serum sodium was 125 mEq/L and serum osmolality was 317 mOsm/kg. We suspected pseudohyponatremia caused by hyperlipidemia and hyperglycemia, and tried normal saline infusion. In spite of significant improvement in glucose level for the first 12 hr, sodium level dropped to 115 mEq/L. After correcting the hyponatremia with 3% NaCl solution, continuous insulin infusion and hydration for next 12 hr, serum sodium level rose to 121 mEq/L.

On the second day of hospitalization, the patient complained of aggravated epigastric pain. Follow-up laboratory analyses revealed a serum amylase level of 443 U/L and a serum lipase level of 615 U/L. On abdominal computed tomography (CT) scan, the pancreas was diffusely swollen with peripancreatic fat infiltration and fluid collection, which suggested acute pancreatitis grade D, according to the Balthazar CT severity index (Fig. 2). Ranson's score was 4 at this point. There was no lipidemia retinalis on an ophthalmologic exam. On the third day of hospitalization, the TG was 506 mg/dL, total cholesterol 281 mg/dL, LDL-C 101 mg/dL and HDL-C 36 mg/dL. The epigastric pain had diminished. The patient commenced oral intake, multiple subcutaneous insulin injection and fibrate medication. On the fourth day of hospitalization, the serum amylase level was 50 U/L and lipase level was 36 U/L. The patient was discharged without any complication on post-admission day 8. Fourteen days after discharge, analyses revealed total cholesterol 308 mg/dL, TG 309 mg/dL, LDL-C 184 mg/dL, HDL-C 66 mg/dL, serum amylase level 107 U/L and serum lipase level 48 U/L. Lipoprotein electrophoresis performed after recovery showed a normal pattern.

97.2 - Choi EY, Gomes WA, Haigentz M, Graber JJ. Association between malignancy and non-alcoholic Wernicke’s encephalopathy: a case report and literature review. Neuro-Oncology Practice. 2016;3(3):196-207. doi:10.1093/nop/npv036

We present the case of a 47-year-old woman diagnosed with laryngeal cancer 3 years prior to admission, which had been treated with chemotherapy (1 cycle of docetaxel, cisplatin, and 5-fluorouracil [TPF]), radiation therapy, and resection (transoral robotic surgery [TORS] and bilateral neck dissection), but found to have recurred several months prior to admission. Her past medical history is also significant for hypertension and end-stage renal disease for which she receives hemodialysis. She had been recently discharged from our hospital 1 week prior to admission, when she was noted to have severe malnutrition as she was not tolerating oral intake. The severity of her malnutrition was illustrated by her rapid and profound weight loss of 43 kg over the course of 6 months. At that time, she refused percutaneous endoscopic gastrostomy tube placement, but recovered her ability to eat food after speech and laryngeal rehabilitation. She returned to our hospital complaining only of nausea, but appeared acutely ill with delirium, and also appeared malnourished.

Initial evaluation revealed bacteremia and her mental condition improved with antibiotics and supportive therapy. She required a prolonged hospital stay, during which time she began to complain of double vision. Neurological examination at that time was significant for persistent, pendular nystagmus in primary gaze and in all directions, as well as jerky ophthalmologic pursuit and slow saccades. After several weeks, while still in the hospital, she had deterioration in her mental status and again developed acute confusion. She also had a newly ataxic gait and impaired short-term memory and alertness.

She had a full evaluation for infection, which was negative, and she remained afebrile and with normal white blood cell count. Her serology, including basic metabolic panel and complete blood count, were unremarkable. Given her recurrent laryngeal cancer, there was high suspicion for CNS metastases and leptomeningeal disease, but lumbar puncture was normal, with unremarkable cytology and flow cytometry. MRI of her brain revealed T2-weighted fluid-attenuated inversion recovery (FLAIR) hyperintensities in the periaqueductal area, medial thalami, and tectal plate, which is typical for Wernicke's encephalopathy (Figs. 1–4)

Given clinical findings and imaging results, she was diagnosed with acute Wernicke's encephalopathy, and she was treated with aggressive thiamine repletion at a dosage of 500 mg intravenously every 8 hours for 5 days. She had immediate improvement in her mental status, became attentive, and was no longer confused. She had persistence, however, of her memory impairment and had poor scoring on recall tasks. There was partial improvement in her nystagmus and ataxia.

She agreed to percutaneous endoscopic gastrostomy tube placement, and was also instructed to take regular, lifetime thiamine supplementation at 100 mg daily via gastric tube. She was discharged to a sub-acute rehab facility, where she regained much of her strength, and was able to ambulate independently for short distances 4 weeks after arrival. She was last seen in our hospital 2 months after discharge, when she was found to have gained 2 kg in body weight.

98.2 - Kadous A, Abdelgawad AA, Kanlic E. Deep Venous Thrombosis and Pulmonary Embolism after Surgical Treatment of Ankle Fractures: A Case Report and Review of Literature. The Journal of Foot and Ankle Surgery. 2012;51(4):457-463. doi:10.1053/j.jfas.2012.04.016

A 66-year-old female patient had sustained a twisting injury to her right ankle while hiking. The injury resulted in trimalleolar ankle fracture dislocation. The patient was transferred by air (a distance of about 130 miles) to a level 1 trauma center. Closed reduction under conscious sedation was performed in the emergency department with application of a posterior splint. The patient was seen 10 days later in the outpatient clinic for a preoperative evaluation. At 13 days after the injury, the patient underwent surgery for open reduction internal fixation of her medial and lateral malleoli. The tourniquet time was 100 minutes. A postoperative splint was applied. The total time for the procedure (with splint application) was 124 minutes. The patient was admitted overnight for pain control, and she was discharged home the next day. No thromboprophylaxis was prescribed. The patient was seen by the physical therapist while in the hospital. She was taught how to ambulate with a walker and was instructed to keep the leg elevated above the heart level as much as possible, to wiggle her toes frequently, and to be non–weightbearing on the affected side (Fig. 1).

Three days later, the patient presented to the emergency department with the complaint of shortness of breath and chest pain. Doppler ultrasonography was performed that revealed DVT of the popliteal and midfemoral veins. At 6 hours after admission to the emergency department, the patient developed massive PE with rapid progression of dyspnea. She was intubated and transferred to the intensive care unit. Shortly after intubation, patient developed bradycardia and hypotension, and cardiopulmonary resuscitation was initiated. She developed anoxic brain injury and acute renal failure. The patient stayed in the intensive care unit for 24 days. A percutaneous endoscopic gastrostomy tube and a tracheostomy tube were inserted, and the patient was discharged to a long-term care facility.

101.2 - Salcin S, Fontem F. Recurrent SARS-CoV-2 infection resulting in acute respiratory distress syndrome and development of pulmonary hypertension: A case report. Respiratory Medicine Case Reports. 2021;33:101314. doi:10.1016/j.rmcr.2020.101314

A 62 year old female with past medical history of previously diagnosed and treated COVID-19 infection, hypertension, hypothyroidism, chronic lower back pain, degenerative disk disease, previous L2-L4 lumbar fusion, and anxiety presented to the emergency department in August 2020 for one week history of progressively worsening shortness of breath and cough. She reported recent contact with a known COVID positive individual. Her grandson had tested positive 10–14 days prior to the onset of her new symptoms. The patient was previously diagnosed with COVID-19 in April 2020. At that time, she was hospitalized and underwent a typical treatment approach based on early understanding of COVID-19 with broad spectrum antibiotics, hydroxychloroquine, vitamin C, and zinc [5]. She recovered and was discharged home with complete resolution of symptoms and no lingering dyspnea or hypoxia. Prior to her COVID-19 diagnosis, the patient had been following with neurosurgery for chronic lower back pain. Planned surgical intervention was delayed due to her SARS-CoV-2 infection. In July, the patient underwent pre-operative clearance for neurosurgical intervention. She was tested for COVID-19 with a resulting negative RT-PCR test on July 16, 2020. She was asymptomatic at that time and had no fever, dyspnea, or hypoxia.

In the emergency department, the patient was tachycardic, tachypneic, and profoundly hypoxic with oxygen saturation as low as 77%. Initial admission arterial blood gas showed worsening hypoxia. She was placed on high flow oxygen at 30 L, 60% FiO2, and admitted to the medical floor. Initial work-up was significant for CTA pulmonary with diffuse bilateral pulmonary opacities suggestive of multifocal pneumonia and ARDS. Comparison of imaging to prior CTA done in April revealed extensive progression of infiltrates (see Fig. 1). Inflammatory markers were significant for sedimentation rate 72, C-reactive protein 10.8, fibrinogen 601, d-dimer 2.035, ferritin 804, and lactate dehydrogenase 508 (see Table 1). She was started on dexamethasone, remdesevir, ceftriaxone, azithromycin, vitamin C, and zinc [6].

Chest radiographs (Panels A and B) taken at time of first presentation to the emergency room show interval development of worsening bilateral pulmonary opacities between April (Panel A) and August (Panel B). Axial, contrast enhanced CT angiography images (Panels C and D) taken at time of first presentation to the emergency room show bilateral pulmonary opacities. Panel D (August) represents development of multifocal pneumonia suggestive of Acute Respiratory Distress Syndrome.

Overnight on day two of admission, she became progressively more hypotensive with increased work of breathing, lethargy, and confusion. Oxygen requirements continued to increase from the previous day to high flow at 40 L, 70% FiO2. At 7am, a rapid response was called. A repeat chest x-ray showed worsening of severe interstitial opacities. The patient was started on BiPAP and pressor support and was transferred to the intensive care unit. Upon admission to the ICU, the decision was made to intubate the patient due to increased work of breathing, progressive hypoxia, and respiratory muscle fatigue. Later that day, her admission COVID test resulted as positive.Three days later, the patient made some interval improvement and was extubated. Unfortunately, she decompensated again and required re-intubation. At time of second intubation, due to the severity of her condition, she was started on convalescent plasma, broad spectrum antibiotic therapy, and a continuous steroid drip. Five days later, she was able to tolerate extubation and was transferred to the medical floor on 6 L of nasal cannula oxygen. Prior to discharge, she tested negative for COVID-19 and went home on 3 L of oxygen.

At her six week follow up appointment, the patient reported continued dyspnea and new lower extremity edema. A trans-thoracic echocardiogram done at this time found newly elevated right ventricular systolic pressures. In order to confirm the diagnosis of pulmonary hypertension, she underwent a right heart catheterization. Catheterization studies demonstrated elevations in pulmonary artery pressure, pulmonary capillary wedge pressure, and pulmonary vascular resistance consistent with the diagnosis of pulmonary hypertension. At this time, she remains on home oxygen with substantial dyspnea and fatigue (Fig. 2).

102.2 - Dietrich A, Mortensen M, Wheller J. Cardiac toxicity in an adolescent following chronic lithium and imipramine therapy. Journal of Adolescent Health. 1993;14(5):394-397. doi:10.1016/S1054-139X(08)80014-6

A 13-year-old male with a history of a complex conduct disorder that had been unresponsive to numerous psychoactive medications was admitted to a pediatric hospital with a 1-month history of progressive fatigue, shortness of breath, chest pain, and edema. Six months previously he was discharged from a psychiatric facility receiving imipramine (225 rag/day) and lithium (2400 rag/day). He had no history of cardiac disease and had a normal physical examination prior to initiation of drug therapy. The patient was healthy except for his psychiatric disease. On admission, his vital signs were as follows: heart rate 120/rain, respiratory rate 40/rain, blood pressure 108/60 mm Hg, and weight 67.7 kg. He was diaphoretic, pale, and in obvious respiratory distress. Auscultation of the chest revealed bilateral rales. The cardiac apex was displaced laterally. There was a loud gallop rhythm. The liver was enlarged and the edge palpable 4 cm below the right costal margin, and there was pitting pretibial edema. A chest radiograph revealed cardiomegaly (CT ratio, 0.7) and diffuse pulmonary edema. An electrocardiogram (EKG) showed low voltage, a P-R interval of 0.20 sec, a QRS duration of 0.10 sec, and T-wave inversion in the left precordial leads (Figure 1). Echocardiography (ECHO) demonstrated a dilated left ventricle (end-diastolic dimension 6.5 cm, normal 5.5 cm.) and a markedly reduced shortening fraction of 18% (normal, 25%-45%). Congestive heart failure was attributed to a cardiomyopathy. No respiratory or gastrointestinal symptoms suggestive of a viral illness had occurred during the previous month. Initial laboratory results induded: normal serum electrolytes, blood-urea nitrogen (BUN), creatinine, Ca, Mg, and glucose; Arterial blood gases (ABG) while receiving mask oxygen pH 7.44, PaCo2 29 torr, PaO2 68 torr; ESR 16 mm/hr (normal); serum imipramine 132 ng/mL and desipramine 44 ng/mL (reported therapeutic levels for both, 150-300 ng/mL); and serum lithium 2.2 mEq/L (therapeutic, 0.5-1.5 mEq/L). Thyroid studies were recommended by the consulting toxicologist and results were as follows: T4 5.7 ~g/dL (normal 5.6-11.7 i~g/dL); T3 resin uptake 45.2% (normal, 37.5%-47.7%) and TSH 12.8 IU/ mL (normal, 0.6-6.5). Multiple viral cultures, viral serologies, and tests for Group A Streptococcus were negative. Therapy included dobutamine, digoxin, furosemide, spironolactone, and fluid restriction. All psychotropic medications were withheld. The consulting endocrinologist interpreted the patient's clinical presentation and thyroid tests as being consistent with borderline hypothyroidism (high TSH, low normal T4), and recommended replacement therapy. During the week after admisison he improved dramatically. His medications included digoxin 0.25 mg q.d., furosemide 40 mg b.i.d., captopril 6.25 mg b.i.d., and levothyroxine 0.1 mg q.d. His weight decreased 10 kg, and, for the first time in over 7 days, he was able to ambulate short distances. A chest radiograph showed a reduced heart size (CT ratio, 0.5) and resolution of the pulmonary edema. ECHO continued to show decreased left-ventricular contractility with a shortening fraction of 20%. There also was mild mitral insufficiency. Because his cardiac status improved rapidly with conventional therapy, endomyocardial biopsy was not considered to be necessary. Holter monitoring was performed for 24 hr on day 10 and showed no ectopy. Holter monitoring on day 17 showed 300 premature ventricular contractions (PVCs) with multiform complexes and one couplet. There was no ventricular tachycardia. Repeat thyroid function tests were normal. Tocainide 200 mg Q8H was started, and 2 days later a repeat Holter monitoring demonstrated only 10 uniform PVCs in 24 hr (essentially normal). He was discharged 23 days after admission on digoxin, furosemide, captopril, levothyroxine, and tocainide. Over the next 24 months he continued to improve and his medications were gradually withdrawn except for levothyroxine and tocainide. Chest radiograph and EKG were normal 13 months after discharge. Holter monitor studies were normal (no ectopy) during follow-up and remained normal after tocainide was discontinued, 14 months after discharge. Three years after discharge and when all his medications had been discontinued, his cardiac examination, EKG, and ECHO were normal, and a 24- hr Holter monitor showed no ectopy. His behavior disorder has remained stable, and he has continued to be difficult to manage.

103.2 - Clancy K, Wong J, Spicher A. Abdominal Aortic Aneurysm: A Case Report and Literature Review. Perm J. 2019;23:18.218. doi:10.7812/TPP/18.218

A 79-year-old man presented to the Emergency Department reporting the acute onset of low back pain radiating to the left side of his chest. The pain started earlier the same day, had become progressively worse, and was not relieved by changes in position. The patient denied hematuria, dysuria, constipation, diarrhea, or any recent trauma.

His medical history was remarkable for known cardiovascular disease, including coronary artery disease with previous myocardial infarction and 4-vessel coronary artery bypass graft; a 31 pack-year history of tobacco use; paroxysmal atrial fibrillation treated with warfarin anticoagulation; and hypertension. Other comorbidities included stage 3 chronic renal insufficiency and hyperlipidemia. He had no known history of aortic aneurysm. On presentation, the patient’s vital signs were stable; however, he appeared in obvious discomfort. His blood pressure was 104/66 mmHg, pulse was 64/min, respiratory rate was 16/min, oxygen saturation was 97% on room air, temperature was 36.5 °C, and body mass index was 28.62 kg/m2. Results of his physical examination included clear lungs; a regular heart rate and rhythm; and an obese abdomen that was soft and had mild distention. No AAA or hepatosplenomegaly were palpated. The patient had considerable discomfort on light palpation of the abdomen, with pain radiating to his flank and back bilaterally.

Laboratory results were overall unremarkable and included a normal troponin I level of 0.01 ng/mL (normal value = 0.00–0.09 ng/mL), white blood cell count of 13.9 × 109/L, and hematocrit of 41%. Of note, the patient’s anticoagulation was subtherapeutic with an international normalized ratio of 1.7 (goal range = 2.0–3.0). The results of an electrocardiogram demonstrated normal sinus rhythm without acute ST changes. The results of an emergent computed tomography angiography (CTA) scan of his abdomen and pelvis demonstrated a 10-cm AAA with a large retroperitoneal hematoma consistent with a contained aortic rupture (Figure 1).

104.2 - Rodriguez-Catarino M, Blimark C, Willén J, Mellqvist UH, Rödjer S. Percutaneous vertebroplasty at C2: case report of a patient with multiple myeloma and a literature review. Eur Spine J. 2007;16(S3):242-249. doi:10.1007/s00586-006-0256-z

A 47-year-old woman was referred to the orthopedic clinic at our hospital with a 6-week history of increasing neck pain without focal neurological symptoms. One night, a week before admission, the pain worsened as she removed a pillow under her neck. She heard a cracking noise, experienced instant pain in her neck and was not able to sustain her head. The X-ray images obtained at her local hospital demonstrated a fracture in C2 and she was referred to us for further investigation. No signs of spinal cord compression or other neurological deficit were found at the physical examination upon admission. Besides the C2 fracture, a CT scan showed an extensive osteolytic lesion (Fig. 1). MR images of the cervical spine showed a prevertebral soft tissue mass and edema (Fig. 2). The patient was given a stiff cervical collar to prevent the development of neurological deficit due to the high risk of subluxation and angulation of the fracture. Osteolytic lesions on the T7, T11 and T12 vertebrae, the 8th and 12th left rib, the iliac bone and smaller lesions in other parts of the hip and in both humeri were demonstrated on the radiological work-up. Several CT-guided biopsies from C2 were inconclusive. Routine laboratory examinations showed normal peripheral blood values, s-electrolytes, including calcium, and s-creatinine. Finally, CT-guided biopsies from the affected ribs and pelvis showed infiltration of plasma cells. In the bone marrow aspirate, 20% plasma cells were found. She had no M-component in serum but showed urinary light chain excretion (3 g/24 h χ-chains) and the diagnostic criteria for multiple myeloma (MM) were fulfilled.

105.2 - Ofluoglu O, Ofluoglu D. A case report: pregnancy-induced severe osteoporosis with eight vertebral fractures. Rheumatol Int. 2008;29(2):197-201. doi:10.1007/s00296-008-0641-5

A 30-year-old woman during the last month of her first pregnancy had moderate back pain. After delivery, her back pain has gotten worse. She was previously diagnosed as myofascial pain syndrome in another medical center and treated with physical modalities (heat application and TENS) and analgesic-myorelaxants. These treatment procedures did not help her pain. Therefore, at 3 months following delivery, she was admitted to the physician with complaints about severe back pain and height loss. She was not able to carry and breast-feed her baby due to severe pain. She could breast-feed her baby for only 1 month because of using pain killers. She had difficulties with her daily activities as well. There was tenderness in thoracic vertebrae and spasm on vertebral muscles. Even though the spinal range of motion was limited, there was no abnormal neurological sign in physical examination. The pain was localized to the back and there was no numbness and/or weakness on lower limbs. She had apparent thoracic kyphosis. Her height was 152 cm, arm span was 164 cm and weight was 50 kg. She reported that her previous height was 161 cm indicating 9 cm height loss.

106.2 - Echahdi H, Hasbaoui BE, Khorassani ME, Agadr A, Khattab M. Von Willebrand’s disease: case report and review of literature. Pan Afr Med J. 2017;27. doi:10.11604/pamj.2017.27.147.12248

We report the 27-month-old man who had been referred due to haemorrhagic shock. Pregnancy and birth history were unremarkable. The boy had been born full-term (39 weeks) with a birth weight (3200g). No parental consanguinity was observed. He was on exclusively breast-fed, there were no incidents during vaccinations, food diversification was started at 6 months old, his weight, length and psychomotor development were within the normal range. He was until yet not circumcised. Prior to the symptoms, the child was described as a good eater, was on a normal diet and was thriving appropriately. The boy was admitted in our department for hemorrhagic shock, he was lethargic, very pale with profound hypotonia, tachycardic, tachypnea and Oliguria at the presentation, the capillary refill time (CRT) was > 3s. Blood was oozing from a wound at the inner surface of the lower lip, the gums appeared otherwise healthy. No other abnormal findings were evident on physical examination (there were no organomegaly and no joint disease (Hemarthrosis). The child had one days before his admission a trauma to the inner face of the lower lip that caused an external acute bleeding loss. The initial step of resuscitation was; immediately tried to stop the source of hemorrhage by manual compression, to restore circulating blood volume by administration of hypertonic salt solutions through large intravenous access calibre with providing an adequate oxygenation. Blood count pronounced: hemoglobin (Hb) level at 3 g/dL; leukocyte and platelet count were normal. Blood chemistry showed: Prothrombin Time (PT) and fibrinogen level were normal while activated partial thromboplastin time (PTT or APTT) was elongated. On the basis of the clinical presentation of hemorrhage, a tentative diagnosis of vWD had been made. This diagnosis was supported by the normal platelet count and the results of the coagulation panel. Following erythrocyte and fresh-frozen plasma (FFP) transfusions, administration of factor VII (Novoseven) was initiated at the admission. Less than 2 hr after transfusion began, the child appeared pinker and the oral bleeding had stopped. Three hours after transfusion, vital signs were within normal limits; the heart rate had decreased to 75 bpm and the respiratory rate was 26 bpm. The rest of biologic data was reported on day 4 and showed normal factor IX level but factor VIII level was low at 16.6%. The result of Factor Von Willebrand: Ag assay was very low at 1.8% that confirmed a severe form of Von Willebrand disease (type 3). The child was discharged with recommendation of relatif sedentary lifestyle in combination with prophylactic therapy in minor surgery including dental work.

108.2 - Roberts SL, Sedley B. Acceptance and Commitment Therapy With Older Adults: Rationale and Case Study of an 89-Year-Old With Depression and Generalized Anxiety Disorder. Clinical Case Studies. 2016;15(1):53-67. doi:10.1177/1534650115589754

During assessment following a call to a crisis line, Margaret reported that her primary concern

was that she had significant and increasing memory loss, which confirmed her belief she had

dementia. She gave examples of not being able to find items in the house, remember recipes,

remember acquaintances’ faces or names, and find her way in less frequented streets, and leave the house without all required items. She also described some word-finding difficulty and poor spelling. Margaret reported testing her memory daily (e.g., if she could immediately find a particular book title on the shelf among the thousands of books in the house), which usually confirmed her assertion that she had Alzheimer’s disease. This fueled worries, both in the present, for example, that she is offending people by not remembering their name, and in the future, for example, that she is “losing her mind.” She began to anticipate that she will end up like her mother who was diagnosed with dementia in her 80s. Margaret was also distressed by her physical functioning, describing nocturnal incontinence, reduced hearing and sight, and pain in parts of her body, and she struggled to accept the need to use mobility aids to assist her. Worries accompanied her physical decline, such as what others would think if she was incontinent in public (although this had not happened). She described being “a silly old woman in a useless body with an even more useless mind” and not liking the person she had become (in older age). Margaret also described feeling lonely and lacking an emotionally reciprocal relationship with her husband who she believed did not empathize with her concerns. He reported that she should “just stop worrying about things related to getting old,” which is what he had done. Although he was pleased that she was seeing a therapist, he declined to be involved in any way. Margaret was resigned that her husband would not change to become a more caring partner as she would like him to. This resulted in feelings of resentment toward him that increased in intensity as she aged and her social circle diminished. Margaret worried about the relationship her husband had with one of her children and believed she needed to stay alive longer than him to make sure this child got their inheritance. She also reported worries about dying before her husband because he may begin a relationship “with a gold digger” leaving no inheritance for any of her children. Margaret also reported concerns about her children. She described lack of emotional support from two of her children who lived locally and she felt were aloof. She described one child who lived overseas as particularly supportive but physically distant. This child expressed concern Roberts and Sedley 57 about her mother’s mental state, particularly her thoughts of murder–suicide, and wrote requesting the therapist ask Margaret to reconsider the role of psychiatric medication in her treatment.

109.2 - Cardoso C, Cremers I, Oliveira AP. Spontaneous bacterial peritonitis caused by Listeria monocytogenes: a case report and literature review. Annals of Hepatology. 2012;11(6):955-957. doi:10.1016/S1665-2681(19)31425-5

A 51-year-old man with alcoholic cirrhosis was admitted to our hospital with a three day history of ascites, peripheral edema and jaundice. The patient denied fever, changes in level of consciousness, vomits, abdominal pain, diarrhea, ileus or gastrointestinal bleeding.

Physical examination revealed a temperature of 36.7°C, no signs of encephalopathy, blood pressure, heart and respiratory rates within normal ranges, jaundice of the skin and sclerotics, a large volume of ascites and peripheral edema.

Blood tests showed:

Hemoglobin 12.7 g/dL (range 13-17).

Leucocytes 13,200/μL (range 4,500-11,400) with 84.8% PMN.

Platelets 130,000/μL (range 150,000-450,000).

Total bilirubin 5.98 mg/dL (range 0.2-1).

International normalized ratio-1.8 (range 0.8-1.2).

Creatinine serum level-1.0 mg/dL (range 0.7-1.3), and

Albumin-2.3 g/dL (range 3.5-5).

A diagnostic paracentesis was performed showing a serum-ascites albumin gradient ≥ 1.1 g/dL with 19,600 leukocytes/μL (absolute neutrophil count of 16,450). A diagnosis of SBP was made and intravenous cefotaxime 2 g every 12 h was promptly prescribed after ascitic fluid inoculation into aerobic blood culture. Despite empiric antibiotic therapy, there was a worsening of peripheral and ascitic fluid white blood cell count at 48 h analytical control.

Three days after the admission, ascitic fluid culture yielded a positive result for Listeria monocytogenes. Cefotaxime was discontinued and the patient started amoxicillin 2.2 g iv every 8 h according to an in vitro sensibility test that had also revealed resistance of this rod to cefotaxime.

However, despite appropriate antibiotics, a rapid clinical and analytical deterioration was observed with the onset of encephalopathy and oliguric renal failure that led to the patient's death 7 days after the admission.

110.2 - Oh RC, Johnson JD. Chest Pain and Costochondritis Associated with Vitamin D Deficiency: A Report of Two Cases. Case Reports in Medicine. 2012;2012:1-3. doi:10.1155/2012/375730

Case 1. A 35-year-old white female presented to the family medicine clinic in Hawaii with complaints of chronic chest pain for the last 3 years. She recently moved from Northern Virginia to Hawaii. Chart review was notable for a negative cardiac workup, including a treadmill stress test, and echocardiogram. Over the last 3 years, the diagnoses of her chest pain included anxiety, esophageal reflux, and costochondritis. In Hawaii, her exam was remarkable only for tenderness to palpation over the left and right costochondral junctions. Since she reported very little milk intake or sun exposure, a serum 25-OH Vitamin D (25-OHD) level was obtained and returned at 42 nmol/L (17 ng/mL), consistent with deficiency. She was started on Vitamin D, 1000 international units (IU) daily. On 3-month followup, her repeat serum 25-OHD was 72 nmol/L (29 ng/mL) and she had complete resolution of her chest pain.

Case 2. A 42-year-old Asian female with a history of carotid Doppler of hypertension and hyperlipidemia presented to a Hawaii emergency department with complaints of substernal chest pain. She was admitted to the hospital for a rule-out myocardial infarction protocol. In the hospital, she was found to have marked tenderness along the left costochondral junction. She did not routinely drink milk and despite living in Hawaii, she reported little sun exposure due to her work hours. After ruling out for myocardial infarction, she was discharged with followup for a treadmill stress test, which was normal. Prior to her discharge, a serum 25-OHD level was drawn to rule out vitamin D deficiency. On followup, her vitamin D level returned 27 nmol/L (11 ng/mL) and she continued to report chest pains. She was started on oral Vitamin D2 50,000 IU once a week for 8 weeks and maintained on 1,000 IU a day thereafter. Repeat 25-OHD level 2 months later was 82 nmol/L (33 ng/mL). Her chest pain resolved with treatment.

115.2 - Lee DC, Swaminathan AK. Sensitivity of Ultrasound for the Diagnosis of Tubo-Ovarian Abscess: A Case Report and Literature Review. The Journal of Emergency Medicine. 2011;40(2):170-175. doi:10.1016/j.jemermed.2010.02.033

A 31-year-old woman presented to the ED with the chief complaint of diffuse abdominal pain for several hours duration. She denied nausea, vomiting, diarrhea, dysuria, or vaginal discharge, and otherwise had a negative gastrointestinal and urologic review of systems. Her last menstrual period was 2 months prior. She had a recent history of irregular menses and one prior elective termination of pregnancy. Her last sexual activity was 7 months prior. The patient was afebrile and hemodynamically stable. The abdominal examination was remarkable for diffuse tenderness to palpation, voluntary guarding, and rebound most pronounced in the left lower quadrant. The pelvic examination was remarkable for cervical motion tenderness and bilateral adnexal tenderness without masses. The white blood cell count was 16.1 million cells per liter with a neutrophil predominance of 90%. Urinalysis was unremarkable and beta-human chorionic gonadotropin was negative. Given the patient's peritoneal findings, the gynecologic consult service was contacted before diagnostic imaging.

The gynecologic consult service evaluated the patient in the ED and, under supervision of the attending gynecologist, a transvaginal ultrasound was performed. The patient's right adnexa was reported as unremarkable and fully visualized. The consulting service was unable to fully evaluate the left adnexa. The initial consult recommendation noted that the ultrasound did not reveal any obvious pathology to suggest a gynecologic etiology for the patient's peritonitis. Thus, the EPs ordered an abdomen and pelvis CT scan with intravenous and oral contrast enhancement to aid in determining the etiology of the patient's abdominal pain.

The patient was immediately started on intravenous ciprofloxacin and metronidazole for a presumed intra-abdominal infection. The CT scan was performed and demonstrated a 7.5 × 9.5 × 6.0-cm complex collection in the pelvis superior to the uterine fundus with obscuration of the left adnexa consistent with a large tubo-ovarian abscess with associated ileus. The patient was admitted to the gynecological service and treated with intravenous gentamicin, clindamycin, and metronidazole for 2 days, and discharged home on oral doxycycline and metronidazole.

116.2 - Jung EJ, Im DH, Park YH, et al. Female with 46, XY karyotype. Obstet Gynecol Sci. 2017;60(4):378. doi:10.5468/ogs.2017.60.4.378

A 17-year-old girl was referred to our clinic for evaluation of primary amenorrhea and delayed puberty. She had no relevant past medical or family history. Her weight was 56 kg, height was 168 cm, and body mass index was 19.8 kg/m2. The patient had small breasts that were developed inadequately (Tanner breast stage 2) and no pubic hair (Tanner pubic hair stage 1). Examination of the genitalia revealed that the patient had normal female external genitalia with an intact hymen and a vagina with a depth of 5 cm (Fig. 1A). Transabdominal ultrasonography and magnetic resonance imaging (MRI) revealed a small uterus, measuring about 15 mm in length; however, neither ovaries or testes were visible. Laboratory evaluation showed markedly elevated levels of follicle-stimulating hormone (65.08 mIU/mL) and very low estradiol levels (less than 5 pg/mL). The serum level of luteinizing hormone was 21.31 mIU/mL and that of testosterone was 3 ng/dL, which was within the normal range for women of that age. A karyotype was obtained and showed a normal male karyotype 46, XY.

120.2 - Voermans NC, Koetsveld AC, Zwarts MJ. Segmental overlap: foot drop in S1 radiculopathy. Acta Neurochir (Wien). 2006;148(7):809-813. doi:10.1007/s00701-006-0754-0

At the age of 34, a healthy female patient presented with left foot extensor paresis and radicular pain. Physical examination showed weakness of left foot extensors and diminished sensation of the lateral side of the left lower leg and foot. Computertomography and caudography revealed a substantial L4–L5 disc herniation with compression of L5 root on the left side. The pain gradually subsided with bed rest, but recurred soon afterwards. Therefore, a partial L4–L5 laminectomy was performed. The pain disappeared after the operation and she recovered well. Foot extensor muscle strength and sensation recovered almost completely.

At the age of 46, she presented with acute severe pain in her back radiating to her left lower leg. It was accompanied by complete extensor paralysis of the foot. Physical therapy did not result in any improvement. At referral to the neurosurgeon six weeks later, she still experienced paralysis of left foot extensors. The pain had gradually subsided, but reappeared when walking. Sensory impairment of the left lateral lower leg and lateral and medial dorsal foot were still present. Bowel and bladder control were normal. Physical examination revealed paralysis of left foot and toe extensors. Plantar flexion of the foot was normal. Sensation was diminished on the medial and lateral side of the dorsal left foot. The left ankle tendon reflex was absent on the left and present on the right side. She had normal knee jerk reflexes. Lasegue’s test was negative on both sides.

MRI of the lumbar spine showed left L5–S1 mediolateral disc herniation with a sequestrum, which had migrated caudally and caused compression of root S1 on the left side (Fig. 1a). L4–L5 segment revealed the results of the previous partial laminectomy, but neither signs of recurrent disc herniation nor signs of L5 root compression (Fig. 1b). An extraforaminal disc prolaps with L5 root compression was also excluded (Fig. 1c). Laboratory investigations ruled out Lyme borreliosis. Nerve conduction studies excluded concomitant peroneal nerve compression. Compound motor action potential of the left anterior tibial muscle was nearly absent, representing severe axonal degeneration of nerve fibres which are traditionally thought to derive from spinal segment L5. Electromyography of left anterior tibial muscle and long extensor hallucis muscles revealed spontaneous activity, also indicating axonal degeneration. Gluteus medius and maximus and gastrocnemius muscles showed spontaneous activity and polyphasic units, reflecting recent axonal degeneration of nerve fibres of spinal segment S1.

Since the paralysis had existed already for six weeks at the time of referral to the neurosurgeon, this neurological deficit was no indication for operation. However, the foot extensor paralysis failed to improve and when the pain recurred four weeks later and persisted in spite of analgesics and physical therapy, an interlaminar discectomy L5–S1 was performed. No evident S1 nerve root compression was observed, but severe fibrosis of the nerve root S1 was encountered, for which neurolysis was performed. Most probably, the herniated disc which had caused compression of root S1 had retracted spontaneously. The pain ameliorated and the patient was referred to a rehabilitation centre for a foot ankle orthesis.
