# Supplementary material for: Assessing ChatGPT 4.0’s test performance and clinical diagnostic accuracy on USMLE STEP 2 CK and clinical case reports
Source: Sci Rep. 2024 Apr 23;14:9330. doi: 10.1038/s41598-024-58760-x (PMC11039662; doi:10.1038/s41598-024-58760-x)
Supplement: Supplementary file 2 — Supplementary Table S1. [file 41598_2024_58760_MOESM2_ESM.docx]

Supplementary table:

|  | Cases from Pre-2021 | Cases from Post-2021 |
| --- | --- | --- |
| Number of cases | 54 | 9 |
| Accuracy in % | 76% | 67% |

*Supplementary table 1: Comparison of accuracy of ChatGPT4.0 based on pre- vs post-2021 case reports.*
